# Rapid and Bihemispheric Reorganization of Neuronal Activity in Premotor Cortex after Brain Injury

**DOI:** 10.1523/JNEUROSCI.0196-21.2021

**Published:** 2021-11-03

**Authors:** Ian Moreau-Debord, Éléonore Serrano, Stephan Quessy, Numa Dancause

**Affiliations:** ^1^Département de Neurosciences, Faculté de Médecine, Université de Montréal, Montreal, Québec, Canada, H3C 3J7; ^2^Centre interdisciplinaire de recherche sur le cerveau et l'apprentissage (CIRCA), Université de Montréal, Montreal, Québec, Canada, H3C 3J7

**Keywords:** hand, neuromodulation, plasticity, recovery, stroke, TMS

## Abstract

Brain injuries cause hemodynamic changes in several distant, spared areas from the lesion. Our objective was to better understand the neuronal correlates of this reorganization in awake, behaving female monkeys. We used reversible inactivation techniques to “injure” the primary motor cortex, while continuously recording neuronal activity of the ventral premotor cortex in the two hemispheres, before and after the onset of behavioral impairments. Inactivation rapidly induced profound alterations of neuronal discharges that were heterogeneous within each and across the two hemispheres, occurred during movements of either the affected or nonaffected arm, and varied during different phases of grasping. Our results support that extensive, and much more complex than expected, neuronal reorganization takes place in spared areas of the bihemispheric cortical network involved in the control of hand movements. This broad pattern of reorganization offers potential targets that should be considered for the development of neuromodulation protocols applied early after brain injury.

**SIGNIFICANCE STATEMENT** It is well known that brain injuries cause changes in several distant, spared areas of the network, often in the premotor cortex. This reorganization is greater early after the injury and the magnitude of early changes correlates with impairments. However, studies to date have used noninvasive brain imaging approaches or have been conducted in sedated animals. Therefore, we do not know how brain injuries specifically affect the activity of neurons during the generation of movements. Our study clearly shows how a lesion rapidly impacts neurons in the premotor cortex of both hemispheres. A better understanding of these complex changes can help formulate hypotheses for the development of new treatments that specifically target neuronal reorganization induced by lesions in the brain.

## Introduction

Moving the hand to grasp an object is associated with modulation of neuronal activity in several cortical areas, including the primary motor cortex (M1) (Schieber and Hibbard, 1993; Kakei et al., 1999) and premotor areas (Tanji et al., 1987, 1988; Cisek et al., 2003; Nakayama et al., 2015). Interestingly, the cortical network supporting unimanual movements extends to both hemispheres, also involving premotor areas and even M1 ipsilateral to the hand (Donchin et al., 1998; Ames and Churchland, 2019; Heming et al., 2019). In the ventral premotor cortex (PMv), for example, many neurons show little selectivity to the hand used, but are rather coding for the location of the target, its shape, or the configuration of the hand to grasp that target (Tanji et al., 1988; Rizzolatti and Luppino, 2001; Michaels and Scherberger, 2018). Although the ipsilateral hemisphere is not specifically driving the production of corticospinal outputs (Soteropoulos et al., 2011; Li et al., 2015), at least some components of the control of unimanual hand movements involve widespread coordination of neural activity across several cortical areas, in the two hemispheres. In this distributed framework, dysfunction or injury in one region is expected to have far-reaching impacts across multiple areas of the ipsilesional and contralesional hemispheres (Grefkes and Fink, 2014; Silasi and Murphy, 2014).

Not surprisingly, there is extensive support that lesions in the brain cause bihemispheric reorganization. However, most studies have used noninvasive imaging methods based on indirect metabolic measures (Ward, 2015; Crofts et al., 2020). To date, direct *in vivo* recording of the impact of brain injury on neuronal activity has revealed that cortical inactivation or lesion profoundly alters processing of sensory information in the two hemispheres (Meyer et al., 1985; Takatsuru et al., 2009; Sweetnam and Brown, 2013; Kokinovic and Medini, 2018), even within minutes after injury (Sakatani et al., 1990; Ding et al., 2011; Mohajerani et al., 2011). Such rapid neuronal changes can be viewed as consequences of the lesion on the network's homeostasis (von Monakow, 1914; Carrera and Tononi, 2014), with a questionable active involvement in motor function at this stage. They are, however, precursors that form the landscape on which subacute plasticity later takes place and, as such, are likely to have profound effects on recovery after brain injury.

Previous studies on rapid neuronal reorganization after brain lesions have been essentially limited to the sensory cortex, conducted in sedated animal preparations, and using passive stimulation. They have left us with a limited understanding of the early consequences of brain injury on neuronal activity associated with the generation of impaired hand movements. This knowledge is particularly timely and could have a far reaching impact, now that noninvasive neuromodulatory approaches, such as transcranial magnetic stimulation (TMS), are being used to favor motor recovery after brain damage (Grefkes and Fink, 2016). Current stimulation strategies are largely based on concepts of network connectivity and interactions. Stimulations are often used to alter the excitability of areas spared by the injury to remotely help reestablish the functional state across the network involved in the generation of movements. The refinement of the hypotheses underlying the development of these treatments would thus greatly gain from a better understanding of the impact of brain injury on neuronal activity across the motor network.

To address some of these issues, we investigated neuronal reorganization associated with the loss of fine control of hand movements in adult macaque monkeys. Motor deficits were induced using reversible inactivation techniques in the hand representation of M1 while continuously recording before and after cortical injury. This allowed us to identify changes in individual neurons in a highly sensitive and powerful way. In the present report, we focused on recordings in PMv, an area known to undergo physiological and anatomic reorganization after brain lesions that cause impairments of hand movements (Dancause et al., 2005, 2006; Murata et al., 2015; Yamamoto et al., 2019).

## Materials and Methods

### Experimental model and subject details

Two adult female rhesus macaques (Macaca mulatta), Monkey M (5.5 kg) and Monkey S (5.7 kg), were used in the present study. All surgical and experimental procedures were performed in accordance with the guidelines set forth by the Canadian Council on Animal Care and were approved by Comité de Déontologie de l'Expérimentation sur les Animaux of the Université de Montréal.

### Surgical procedures

Surgical procedures were conducted under sterile conditions. Anesthesia was induced with ketamine hydrochloride (15 mg/kg; Ketaset; Pfizer) and maintained with ∼2%-3% isoflurane (Furane; Baxter) in 100% oxygen. Monkeys were given atropine (atropine sulfide; 0.04 mg/kg; Rafter 8 Products) and dexamethasone 2 (Dexacort 2, 0.5 mg/kg; Rafter 8 Products) as well as an intravenous injection of mannitol 20% (1500 mg/kg; Fresenius Kabi Canada) to prevent inflammation and swelling of the brain. Lactated Ringer's solution was injected continuously to maintain hydration (10 ml/kg/h, i.v.). Body temperature was kept near 36.5°C with a self-regulating heating blanket (Harvard Apparatus), and blood oxygen saturation and heart rate were monitored throughout the procedures.

Muscles were intramuscularly implanted with insulated, multistranded microwires (Cooner Wire) for recording of EMG signals. Each microwire was tunneled subcutaneously from the target muscle to a connector embedded in bone cement on the top of the head. Accurate placement of the EMG wires was tested by electrical stimulation of the muscles with the implanted wires and observation of the evoked movements, both during the surgery and in later sessions in the awake state, while the monkey was sitting quietly. We implanted the deltoideus, biceps brachii, brachioradialis, palmaris longus, flexor carpi ulnaris, flexor carpi radialis, flexor digit communis, extensor carpi ulnaris, extensor carpi radialis, extensor digit communis, first dorsal interosseus, adductor of the thumb, and abductor of the thumb. Only channels with clear EMG signals for all selected recording sessions were kept for analysis (see Fig. 3). In the present set of analyses, the EMG signals were used primarily to control for the appearance of covert movements after inactivation.

**Figure 1.**
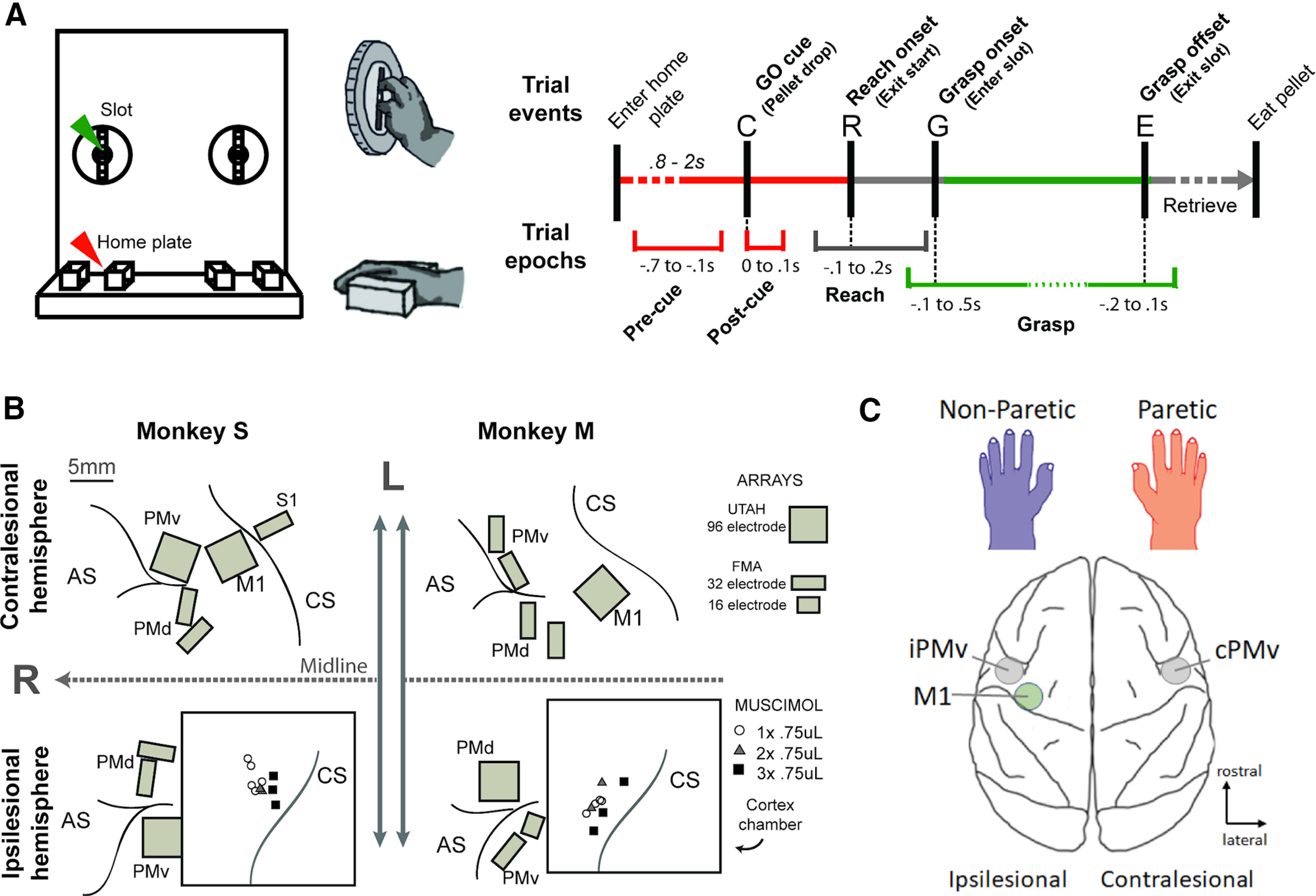
Experimental design. ***A***, Illustration of the task (left). To initiate trials, the monkey placed the hand in the home plate (red arrowhead; bottom right inset). After a variable delay, a pellet was delivered in a distribution slot (green arrowhead) containing a food well (top right inset). Trial events and epochs (right). The home plates and slots were equipped with infrared LED sensors that reported trial events used to define trial epochs for neuronal analyses (see Materials and Methods). ***B***, Reconstruction of the location of the electrode arrays in the 2 monkeys relative to the sulci and the location of the chamber (black square). The muscimol injection sites for the different experiments are shown in the chamber. AS, Arcuate sulcus; CS, central sulcus; L, lateral; R, rostral. ***C***, Schematic representation of iPMv and cPMv, and the paretic and non-paretic hands in relation to the location of the inactivation, which was always in the left M1.

Craniotomies and durectomies were performed to expose M1 and the lateral premotor cortex in both hemispheres. Multielectrode arrays were implanted in the left PMv and dorsal premotor cortex (PMd) and the right PMv, PMd, the M1, and primary somatosensory cortex (S1) (see Fig. 1*B*). In the left hemisphere, the dura was left intact over the central sulcus and a chronic chamber (2 × 2 cm opening; Plexiglas) was positioned to provide access to the dura over the M1 hand representation. The chamber was used to perform muscimol inactivation (see next section) in the left hemisphere, which we refer to as the “ipsilesional” hemisphere. Consequently, the right hemisphere was opposite to the lesion, and referred to as the “contralesional” hemisphere in both monkeys. With this configuration, the right hand was the affected or “paretic” hand.

The placement of the arrays was guided by anatomic landmarks, including the arcuate and central sulcus. In Monkey M, 370 electrodes were implanted. In the ipsilesional hemisphere, one 96-electrodes Utah array (Blackrock Microsystems) was placed in PMd (ipsilesional PMd [iPMd]), and one 32-electrodes and one 16-electrodes floating microprobe array (FMA; Microprobes for Life Science) were placed in PMv (ipsilesional PMv [iPMv]). In the contralesional hemisphere, one 96-electrodes Utah array was placed in M1 (contralesional M1 [cM1]), as well as four 32-electrodes FMAs, two each in PMv and PMd (contralesional PMv and PMd; cPMv and cPMd, respectively). In Monkey S, a total of 448 electrodes were implanted. In the ipsilesional hemisphere, one 96-electrodes Utah array was placed in iPMv, and two 32-electrodes FMAs were placed in iPMd. In the contralesional hemisphere, two 96-electrodes Utah arrays were placed, one in cM1 and one in cPMv, and two 32-electrodes FMAs were placed in cPMd and one in S1. Signals from arrays implanted in iPMv and cPMv were used for the present set of analyses.

At the end of each surgery, monkeys were given Baytril (5 mg/kg; enrofloxacin, Bayer) to protect against infection, another injection of dexamethasone 2 (0.5 mg/kg; Dexacort 2, Rafter 8 Products) to prevent brain swelling, as well as carprofen (4 mg/kg; Rimadyl, Zoetis Canada) and buprenorphine (5 µg/kg; Temgesic, Schering-Plough) to prevent inflammation and pain, respectively. Additional doses of Baytril and Carprofen were given for 2 d after the surgery or longer, following the recommendations of the veterinarian.

### Behavioral task

The monkeys were brought to the laboratory to conduct the neuronal recording sessions. They sat in a custom-made primate chair placed in front of a pellet retrieval task (see Fig. 1*A*, top). The chair had an opening in front of the mouth and removable panels on both sides to allow the use of one hand or the other in different blocks of trials. The pellet rewards (190 mg Dustless Precision Pellets; BioServ) were delivered in a target consisting of a well behind a vertical slot (1.3 cm × 5.5 cm) that was positioned ∼10 cm below the shoulder height and 20 cm from the monkeys. To get the rewards, the animals had to reach with either the left or the right hand and use a precision grip (opposition of the thumb and index) with the forearm pronated. Video recordings of the behavioral task were collected using two computer webcams placed above and on the left side of the animal. The task was controlled by a Tucker-Davis Technologies acquisition system using two RZ2 BioAmp processors and custom software designed for this experiment.

The hand used was determined by which side panel on the chair was removed. Each trial began when the animal placed the hand on the home plate located in front of them, 15 cm below the target. The home plate contained an infrared laser sensor, which detected the presence of the hand. Trial progression is shown in Figure 1*A* (right). After a variable delay period (0.8-2 s), a pellet was delivered into the well by a pellet distributor (80209 Pellet Dispenser, Campden Instrument). The clicking sound associated with the pellet delivery served as GO cue. The animal then had 2 s to perform a self-paced reach toward the target. The movement of the hand out of the home plate was signaled by the sensor and marked the onset of reach. The animal entered the target to grasp the pellet. A second infrared laser placed in the slot containing the food well signaled the hand entering and leaving the target, which were used to mark the onset and offset of grasp. After grasp, the monkey brought the pellet to its mouth, before placing its hand back in the start position to initiate the next trial. The intertrial interval was 3 s.

In each recording session, the monkeys performed 25 trials with the left hand and then 25 trials with the right hand. In inactivation experiments (see below), additional recording sessions were conducted to collect blocks of trials at different time points after the inactivation, always in the same order. Each recording session took ∼10 min to perform.

### EMG recording and analysis

The EMG signals were recorded using a Tucker-Davis Technologies acquisition system. The continuously recorded raw EMG signals were sampled at 4.069 kHz, and separated into individual trials using a custom-built software, before being further analyzed using custom code written in MATLAB (The MathWorks). For each recording session, we took a rectified mean of the activity across a block of trials during use of a given hand, aligned on different task epochs (see below for epoch descriptions). The mean EMG signals were then low-pass filtered at 50 Hz using a second-order Butterworth filter.

To quantify potential changes of EMG after inactivation, we normalized the mean rectified and filtered activity for each muscle using the 500 ms period before the onset of movements for a given block of trials (from −0.6 to −0.1 s from reach onset). The area under the curve of the mean and rectified EMG trace was extracted with data aligned on grasp onset (−0.1 to 0.5 s from event), and on grasp offset (−0.2 to 0.1 s from event). Values of all muscles in one arm for a given block of trials were averaged (e.g., with non-paretic arm, Pre-inactivation). Two paired *t* tests were used to compare the Pre-inactivation to the Post-inactivation blocks of trials: one test with data aligned to grasp onset and the other with data aligned to grasp offset. This was done for EMG data in both the moving and the resting arm. Recordings from all inactivation experiments were included in these analyses (see below).

### Muscimol inactivation experimental procedures

In the first two recording sessions after the implantation of cortical arrays and chamber, we confirmed the location of the hand representation in left M1 with intracortical microstimulation trains. A custom-made borosilicate glass-coated tungsten electrode was lowered within the chamber, perpendicular to the dura, while the animal was sitting quietly. Trains were delivered at 1 Hz, and each train consisted of 13 monophasic cathodal pulses of 0.2 ms at 350 Hz (Dancause et al., 2008). We included in the hand representation all cortical sites from which digit, wrist, or forelimb (i.e., pronation/supination) movements were evoked and located its borders. At the rostral and medial borders, proximal movements were evoked (i.e., elbow or shoulder). At the lateral border, movements of the face were evoked (e.g., lip, tongue, or pinna). The caudal border was located deep in the central sulcus, at cortical sites that did not evoke movement with low stimulation intensity (i.e., ≤ 30 µA). For cortical sites within the hand area, we identified the depth at which the lowest stimulus intensity was required to evoke a movement. These sites and depths were used to guide the muscimol injections.

A total of 13 inactivation experiments were included in the present analyses. Six of these experiments were conducted in Monkey M, and 7 in Monkey S. For most experiments (*n* = 9, 4 from Monkey M, 5 from Monkey S), we did one injection of 0.75 µl of the GABA agonist muscimol (5 mg/ml) in the hand area of the left hemisphere. In each monkey, we also included one additional experiment in which we injected muscimol at two sites (2 × 0.75 µl) and one other experiment in which we injected muscimol at three sites (3 × 0.75 µl) in the hand area (see Fig. 1*B*).

For each inactivation experiment, we first recorded baseline behavioral performance and neural data in a “Pre-inactivation” data collection session. Animals performed a block of trials (*n* = 25) with the left hand followed by a block with the right hand. Then, the M1 hand representation was reversibly inactivated with muscimol, delivered with a 5 µl Hamilton syringe with a beveled 26-gauge needle (Hamilton). The syringe was positioned in the brain using a micromanipulator (David Kopf Instruments) with stereotaxic frame mounted to the primate chair. At each targeted cortical site, 0.75 µl of muscimol was injected at a rate of 4 nl/s with a microinjector (Harvard Apparatus) at different depths adjusted based on intracortical microstimulation data (range ∼4.5-7 mm from top of the dura). Thirty minutes after the injection of muscimol, the animal was required to perform two more blocks of trials (*n* = 25 trials), first with the left hand and then with the right hand, in a second recording session (Post 0.5 h). The animals showed some clear deficits (see Results) while still being able to perform some trials successfully. Notably, neural activity was recorded continuously throughout the Pre-inactivation session, the injection of the muscimol, and the Post 0.5 h session. After the Post 0.5 h recording session, the monkeys were returned to the home cage, where they could move freely and have unlimited access to water.

To follow the progression and extent of motor deficits, monkeys were brought back to the laboratory to record additional sessions 3 h (Post 3 h) and 10 h (Post 10 h) after the inactivation. At Post 3 h after inactivation, the impairments had progressed to the point that monkeys were not able to perform the task with the paretic hand (see Results). To evaluate behavior, the experimenter presented food morsels in front of the monkey with large forceps held vertically, which led the monkeys to attempt reaches and grasping with the forearm in pronation. Deficits were milder at Post 10 h and no longer visible on the following day, confirming the transitory effect of the pharmacological manipulation. We always had a minimum of 72 h between subsequent inactivation experiments.

### Neural recordings and identification of neurons

We focused the present neuronal analyses on Pre-inactivation and Post 0.5 h recording sessions for two main reasons. First, at this time point, the monkeys were still able to successfully perform the task, at least in some trials. This allowed us to compare the neuronal activity in a similar behavioral context, before and after the inactivation. Second, since neuronal activity was recorded continuously from Pre-inactivation to Post 0.5 h, this allowed us to unequivocally follow individual neurons throughout these sessions and to perform “within-neuron” analyses (see below), in which we quantified the changes of activity caused by the inactivation in the discharge pattern of clearly isolated and continuously recorded neurons. To increase the number of neurons in the control condition, we included sessions in which the monkey performed the task, but no inactivation was induced (i.e., Unmanipulated sessions).

We simultaneously recorded neural activity from 256 channels, the maximum possible count with our equipment. In different experiments, we alternated the areas/arrays we focused on. Recordings included in the present analyses are from sessions that prioritized recordings from the arrays implanted in PMv. Neuronal data were sampled at 24,414.1 Hz, bandpass filtered between 100 and 5000 Hz, and recorded digitally using a Tucker-Davis Technologies acquisition system. An automatic threshold (×4 SDs above the baseline noise) was used on all channels that was locked in place for the duration of the recording. When this threshold was crossed, a 1228 µs sample was recorded. These suprathreshold waveforms were sorted offline using principal component analyses in Plexon Offline Sorter (Plexon). Waveforms from a given unit were categorized as “well-isolated” when the waveform cluster was clearly separated from other recorded signals using the first three principal components. Only well-isolated units were included in the current analyses. Then, we verified the stability and isolation of the principal components of each unit for the entire time of recording by plotting the first 2 principal components over time. For the within-neuron analyses (see below), we discarded any unit that completely disappeared or appeared after inactivation or for which the PC1-PC2 cluster isolation in relation to other signals was lost at any point during recording. Thus, although some neurons in these analyses had very few spikes during reaching and grasping in post-inactivation trials (e.g., see Fig. 5*F*), they were sufficiently active during intertrial intervals or between blocks of trials to follow their isolation from the beginning to the end of recording. It should be noted that this approach could have resulted in the removal of neurons that truly shut off or started discharging after inactivation and consequently to an underestimation of the number of neurons with lower or higher firing rate after the inactivation. However, we preferred this potential underestimation to an overestimation of the impact of inactivation. Initial sorting was done by one experimenter (I.M.-D.) and the quality of the isolation of sorted waveforms confirmed by a second (S.Q.). After sorting, we confirmed that units had no refractory period violation (0%) using an interspike interval of ≤1 ms, before and after inactivation.

We ensured that most neurons included in the study were sampled only once in iPMv (66 of 94; 70.2%) and in cPMv (103 of 138; 74.6%) for within-neuron analyses. For this subpopulation, only signals from a single inactivation experiment were selected for any given electrode (i.e., 0% chance of double sampling). We verified that all analyses using “within-neuron” data gave similar results with this subpopulation of neurons. However, because muscimol could potentially affect the activity of a given neuron differently (e.g., depending on the specific location of the injection in relation to the hand representation or the level and nature of impairment it caused), we considered each inactivation experiment as an independent sampling. We thus included signals from more than a single recording session for some of the electrodes in iPMv and cPMv in the total population of neurons (see Results).

### Task epoch modulation of individual neurons

To give an appreciation of the discharge pattern of the total population of neurons, we produced heat plots of discharge rates in relation to the onset of grasp (see Fig. 4*A*,*B*). In these plots, for each neuron the spike density estimate (SDE) of spike discharge at each moment in time was divided by the average SDE across the time window of interest (−1 to 1 s around Grasp Onset) to highlight when neurons had their peak discharge. To characterize if neurons were modulated during one or several epochs of the task, we compared the average firing during the pre-cue epoch (0.7 to −0.1 s before the Go cue), which served as a baseline of neural activity, to the firing rate during other epochs of the task using two-tailed *t* tests. The different epochs of interest, in chronological order, were pre-cue, post-cue, reach, and grasp. The post-cue epoch was the first 0.1 s following the GO cue, and the reach epoch ranged from −0.1 to 0.2 s from Reach Onset, when the monkey's hand left the home plate. The current set of analyses mostly focused on the grasp epoch, which ranged from −0.1 s before the fingers entered the slot (Grasp Onset) to 0.1 s after the fingers had left the slot with the pellet (Grasp Offset). Since the grasp epoch encompasses both the Grasp Onset and Grasp Offset events, the *t* test was performed on spike times aligned on each event separately for some analyses. The two windows of time used were −0.1 to 0.5 s from Grasp Onset and −0.2 to 0.1 s from Grasp Offset. A neuron whose firing rate was modulated according to the *t* test (*p* < 0.05) within either of these windows was considered tuned to grasp during that block of trials. To visualize the modulation of individual neurons during the task, in addition to raster plots, we calculated SDEs of the firing rate across trials for each neuron (Nawrot et al., 1999), with spike times aligned at Grasp Onset (see Fig. 5). The SDEs were performed using a Gaussian kernel function with a kernel of 50 ms.

### Effect of inactivation on the total population of recorded neurons

To explore changes in the general discharge of neurons when the animals were at rest, we looked at the firing rate of control and Post 0.5 h PMv neurons during the pre-cue epoch (−0.7 to −0.1 s from cue) when the animal waited with either hand in the start position. For each neuron, we determined the mean firing rate during the pre-cue epoch for each block of trials (right or left arm, pre or post 0.5 h inactivation). We compared the proportion of neurons modulated during grasp (i.e., identified with the two-tailed *t* test; see above) before and after inactivation (see Fig. 6*A*,*B*) and the proportion of neurons modulated in function of the hand used (see Fig. 6*C*,*D*). All available control and Post 0.5 h neurons were used for these comparisons. When performing these analyses, “control” always included PMv neurons from both hemispheres pooled together, using their activity during ipsilateral or contralateral hand movements, as appropriate. For other analyses, we only used neurons that remained clearly isolated throughout the inactivation experiments, both before (Pre) and Post 0.5 h after inactivation (“within-neuron”; see below).

### Quantification of changes within continuously recorded neurons before and after inactivation (“within-neuron” analyses)

To take into account differences in grasp duration within a given trial block, but also following inactivation, we normalized the duration of grasp for our “within-neuron” analyses (see Figs. 7–11). The SDE of each trial was resampled, attributing a value of “0” at the time of Grasp Onset and “100” at the time of Grasp Offset, such that time is visually represented as percentage (%) of grasp completed. In this normalized time, each time bin represents 1/1000th (0.1%) of grasp duration. The mean trial SDE during the pre-cue epoch for a block of trials served as a baseline, which was then subtracted from the resampled SDE curves.

#### Incidence of neurons with increases and decreases of discharge rate during grasp

We were first interested in determining how many neurons increased or decreased activity during grasp following inactivation (see Fig. 7). For each neuron, and at each moment in normalized time, we determined whether there was an increase or decrease of neural discharge following the inactivation by subtracting the Post-inactivation SDE values from the Pre-inactivation SDE values at that moment in time. We also performed unpaired *t* tests on the SDE values obtained in trials performed before and after inactivation. This provided counts of neurons whose discharge increased or decreased at each moment during grasp and for how many neurons these changes were substantial (i.e., “significant” change reported by the *t* test). Furthermore, to provide an alternative quantification of increases or decreases of neural activity throughout grasp, we calculated cumulative sums of neurons with higher and lower firing rates after inactivation at each moment in time according to the following formula:

(1) $$Cumulativesum=\overset{n}{\underset{i=1}{\sum }}\frac{Neuronshigher\left(i\right)-Neuronsslower(i)}{Totalneurons\times 1200}x100$$

Where *i* is the time bin ranging from 1 to the total number of time bins per trial considered for this analysis, *n* = 1200 (1000 bins for grasp duration, plus the last 100 bins before grasp onset, and the first 100 bins after grasp offset). *Total neurons* represent the number of neurons included in the population, which could either have higher (*Neurons higher*) or lower (*Neurons lower*) firing rates after inactivation compared with Pre-inactivation values, at time *i*, regardless of whether changes were significant or not. Accordingly, values are expressed as a percentage of the maximum sum of neurons with increase possible (i.e., if only increases would occur across the population throughout grasp). Positive values report that the proportion of neurons with higher activity surpassed the proportion of neurons with lower activity as grasping progressed. This reflects an increase of activity in the neuronal population after inactivation. Negative values report that a greater proportion of neurons with lower activity cumulated along grasp and reflects that the neural population became less active after inactivation. To better visualize the effect of the inactivation on the cumulative traces compared with chance, we used Monte Carlo methods. For each neuron, the firing rate at each time bin in the Pre- and Post-inactivation blocks of trials was pooled. Two new artificial SDE traces were generated by randomly selecting data points from this pool of bin values, with replacement. Then, we subtracted the two artificial SDE traces to calculate the increase or decrease of firing rate at each time bin. The same procedure was done for all neurons in a hemisphere and for a block of trials (e.g., iPMv, nonparetic hand trials) and the cumulative sum of this population was calculated. This process was repeated 1000 times. The mean and 95th percentile from all these artificial traces are shown in relation to real values in Figure 7.

#### Changes of peak discharge time and burst rate

For both the peak discharge time (see Figs. 8 and 9) and rate (see Figs. 10 and 11), we wanted to investigate further the changes of activity in neurons that specifically had a burst of activity during grasp (in contrast to neurons modulated according to the *t* test). Because of the time devoted to each epoch, the *t* test is insensitive to neurons with either low spiking rate or with discharge burst of short duration. Therefore, for these analyses, we opted to include neurons that had a maximal discharge value >1 SD from baseline (i.e., pre-cue epoch) in the grasp epoch of the normalized SDE curves, either before or after inactivation. For each neuron with such a burst during grasp, we identified the time of maximal discharge rate during grasp before and after inactivation (see Fig. 8*C*,*D*) and quantified the change by subtracting the two values (see Fig. 9*B*).

For the analyses of burst rate, since neurons could have peaks at different moments during the grasp epoch before and after inactivation, we characterized the amplitude and time of the peak in both recording sessions, when present. For each neuron, we compared the firing rate at the time of the peak for pre-inactivation (i.e., Pre Peak; see, e.g., Fig. 10*A*) to the firing rate at the same moment in normalized time in Post 0.5 h and averaged the changes across the population of neurons. This approach highlights the change of activity at the time when the neuron was most active before the inactivation and emphasizes decreases of neural activity. Similarly, we took the time of peak discharge in Post 0.5 h (i.e., Post 0.5 Peak; see, e.g., Fig. 10*C*) and compared the firing rate of each neuron in the pre-inactivation to the Post 0.5 h at this time and averaged changes across the population. This highlights the change of activity at the time when the neuron was most active after the inactivation and emphasizes increases of neural activity.

Finally, we verified that neural changes followed the time course of behavioral impairments induced by the inactivation (see Fig. 11). We selected well-isolated neurons with a significant burst during grasp with the non-paretic hand and with consistent waveform characteristics across recording sessions for a given inactivation experiment (i.e., Post 0.5 h; Post 3 h and Post 10 h). These analyses were exclusively conducted with trials of the non-paretic hand because monkeys were not able to complete trials with the paretic hand in at least one of the recording sessions. We compared the peak amplitude of neurons in the different sessions after inactivation to the pre-inactivation values, similarly to what was described above. For each neuron, we calculated the mean of the SDE values of trials of a given session in a 1200 bin window that spanned across grasp duration (1000 bins for grasp duration, plus the last 100 bins before grasp onset, and the first 100 bins after grasp offset). Complimentarily, we also performed the same analysis using the discharge amplitude of the population at the time of maximal discharge before inactivation (see Fig. 11*D*).

### Statistical analysis

#### Quantification of muscimol effects on movement duration and EMG

**Figure 2.**
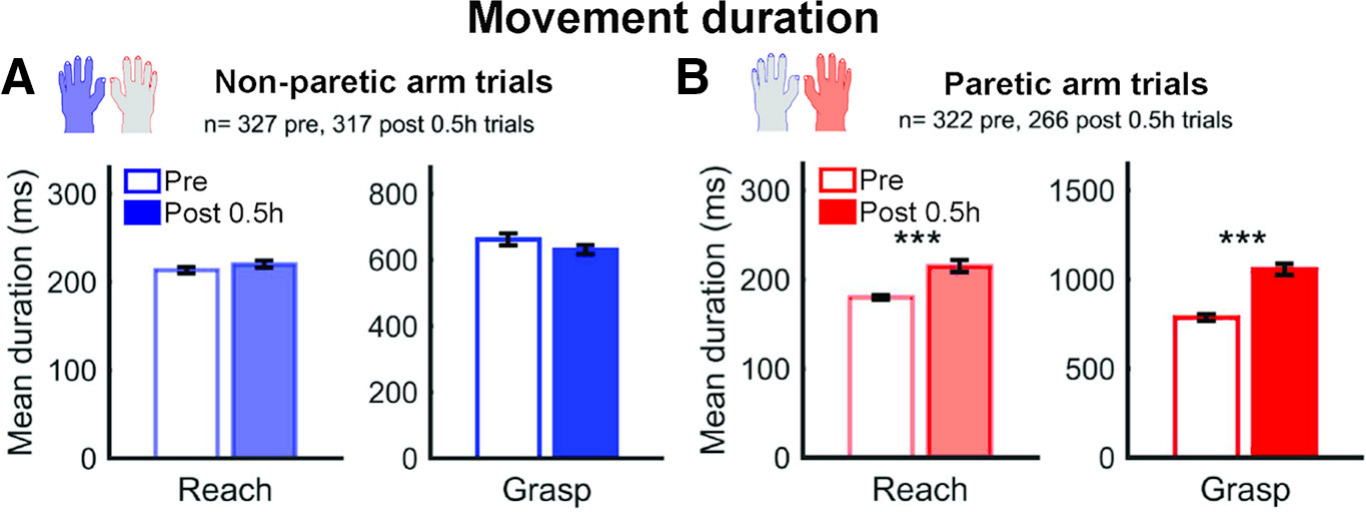
Impact of the inactivation on movement duration 0.5 h after injection of muscimol. ***A***, ***B***, Average reach and grasp duration (±SEM) before (Pre) and 0.5 h after injections of muscimol (Post 0.5 h) during movement of the non-paretic (***A***) and paretic arm (***B***). Data from all sessions for both monkeys are combined. While no changes were observed with the left, “non-paretic hand” (blue bars), both reach and grasp duration were increased with the right, “paretic hand” (red bars). ***p < 0.001 (unpaired *t* tests).

**Figure 3.**
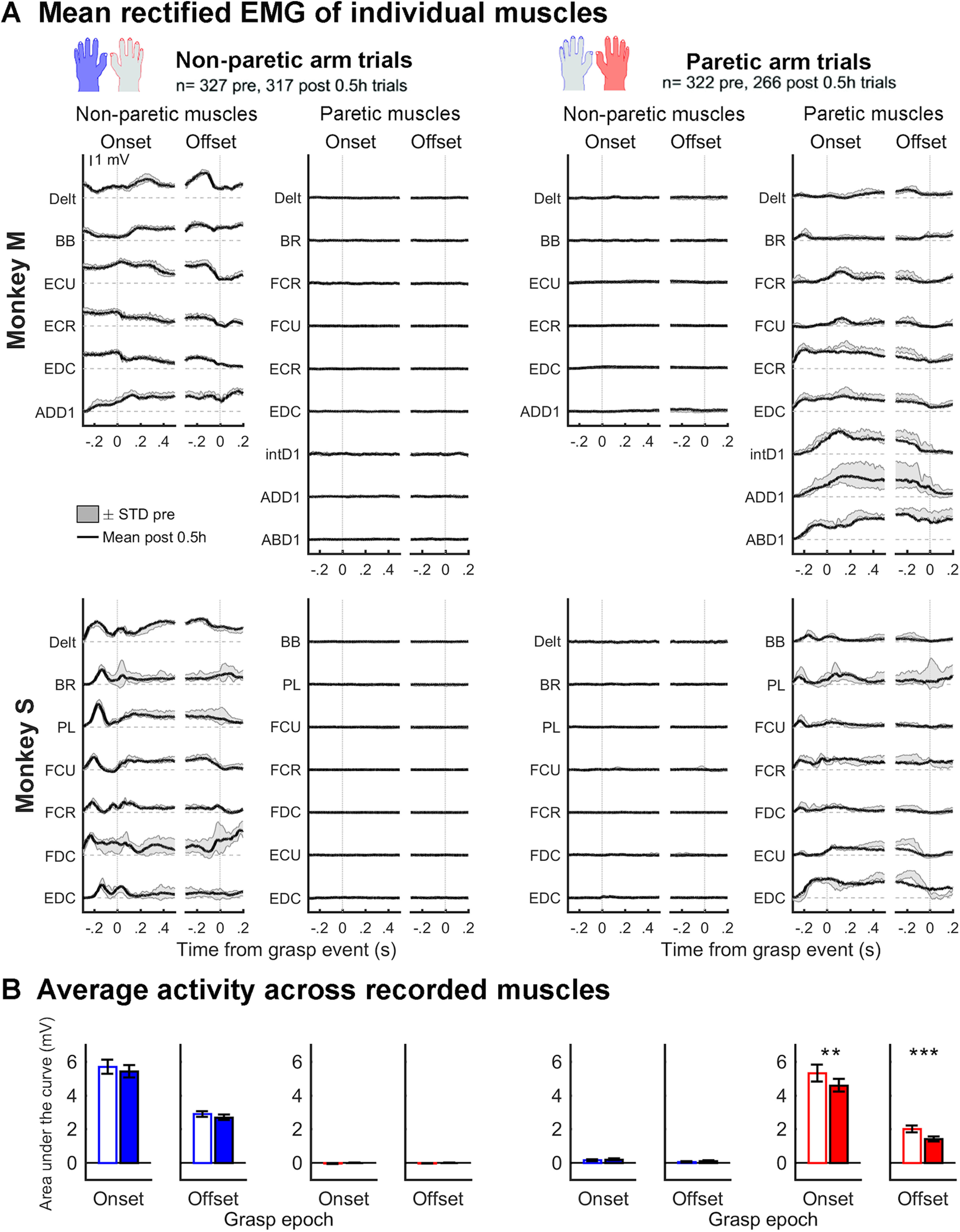
Impact of the inactivation on EMG activity 0.5 h after injection of muscimol. ***A***, Mean rectified and filtered EMG traces in the various muscles recorded during grasp with the non-paretic arm (left) and the paretic arm (right) in Monkey M (top) and Monkey S (bottom). In each plot, EMG traces are shown aligned on Grasp Onset and Grasp Offset using the same time widows used for the neuronal analyses. For each monkey, data from successful trials across inactivation experiments are combined. For each muscle, the average EMG trace of trials collected after inactivation (Post 0.5 h; black line) is overlapping the SDs from the mean of trials collected before inactivation (shaded gray). ***B***, Quantification of EMG changes after inactivation using the area under the curve of the average EMG trace (+/- SEM) across recorded muscles. During trials with the non-paretic arm (left plots), there were no significant changes of EMG activity in the moving arm (i.e., non-paretic arm, blue bars) after inactivation. The error bars show standard errors. This was true both at the onset and the offset of grasp. Little EMG activity was present in the resting arm (i.e., paretic arm), and the inactivation did not cause any changes. During trials with the paretic arm (right plots), there was little activity in the non-paretic arm (i.e., resting arm), both before and after the inactivation. However, there was a significant decrease of EMG activity in the paretic arm (i.e., moving arm; red bars), both at the onset and the offset of grasp. **p < 0.01. ***p < 0.001.

The effects of muscimol on movement duration were evaluated using unpaired *t* tests (see Figs. 2 and 3). For each arm, one test compared the duration of the reach period and one test compared the duration of grasp (*p* < 0.05). To compare the area under the curve of average EMG signals before and after muscimol injections, we used paired *t* tests (*p* < 0.05). Cohen's *d* was used to measure effect sizes when appropriate. In some cases when the sample was too small (i.e., *n* < 20), we used Hedges' *g* to measure the effect size.

#### Quantification of muscimol effects on the total population of recorded neurons

To identify neurons significantly modulated during one or several epochs of the task, we used paired *t* tests (*t* test *p* < 0.05). The discharge of neurons at rest, when the animal waited with the hand in the start position using a 600 ms window before Cue onset (−0.7 to −0.1 s), were compared using a three-way ANOVA (*p* < 0.05), using area (iPMv, cPMv), arm (non-paretic, paretic), and session (control, Post 0.5 h) as factors. The effect size for ANOVAs was estimated using the partial η squared (η*_p_*^2^). Finally, the proportion of neurons modulated during grasp or in function of the hand used was compared using χ^2^ tests (*p* < 0.05) followed by *post hoc* two-proportion *Z* tests with Bonferroni correction (see Fig. 6). The effect size for the χ^2^ tests and *Z* tests was calculated using Cramer's *V* and the correlation coefficient *r* (*r* = z/sqrt(*n*1 + *n*2)), respectively.

#### Quantification of changes within continuously recorded neurons

We compared the variance of peak discharge time after inactivation to control neurons recorded for ∼1 h using Bartlett's test, followed by *post hoc* two-sample *F* tests with Bonferroni correction (see Fig. 9). Comparison of spike discharge rate before and after inactivation was done using paired *t* tests (see Fig. 10).

For the progression of changes with time after muscimol injection (see Fig. 11), we evaluated the effect of time and cortical area (iPMv and cPMv) with two-way repeated-measures ANOVAs, one for mean spike firing rate during grasp (see Fig. 11*B*) and one for discharge amplitude of neurons at the time of their maximal discharge (see Fig. 11*C*,*D*). Since in both cases, there was no effect of area nor interaction between area and session, we merged data from the two hemispheres. Values from each post-inactivation session (i.e., Post 0.5 h; Post 3 h and Post 10 h) were then compared with pre-inactivation using a *t* test, and the *p* value adjusted using a Bonferroni correction (*p* < 0.017).

## Results

We trained 2 adult female rhesus macaques on a reach-to-grasp task (Fig. 1*A*), in which monkeys retrieved food pellets using precision grip with the right or the left hand. Both animals were implanted with chronic electrode arrays in PMv of the two hemispheres (Fig. 1*B*). In addition, a chamber was placed over the left M1, giving access to the dura. After implantation, we located the hand representation in the chamber using intracortical microstimulation trains before initiating inactivation experiments. In each inactivation experiment, we first recorded the neuronal activity during grasping before inactivation (i.e., recording session Pre). Then, the GABA-A agonist muscimol was injected in the hand area of the left M1 through the chamber to induce a reversible inactivation (see Materials and Methods). With this design, the left PMv was always in the same hemisphere as the “lesion” (i.e., “ipsilesional” PMv or iPMv) and the right PMv was in the opposite hemisphere (i.e., “contralesional” PMv or cPMv) (Fig. 1*C*). The monkey resumed performing trials ∼30 min after the injection of muscimol (i.e., session Post 0.5 h). Recordings were uninterrupted throughout these different steps, allowing us to quantify the effect of inactivation on individual neurons.

As previously reported when using comparable inactivation techniques (Matsumura et al., 1991; Schieber and Poliakov, 1998; Brochier et al., 1999), both monkeys showed clear impairments with the contralateral, right hand, which we refer to as the “paretic hand.” In session Post 0.5 h, animals were still able to perform some trials on the task with the paretic hand (minimum = 5, maximum = 25; average ± SD =21.4 ± 5.5), albeit with clear impairments. Examples of deficits included difficulty to oppose the thumb to the index and a reduction of individuated movements of the index (e.g., D2 and D3-5 being moved together), increased number of digit flexions to grasp the pellet, and abnormal coordination between the hand and forearm. Reaching and grasping movements with the paretic hand became slower at Post 0.5 h (*t* test: reach *T*_(586)_ = 5.15, *p* = 3.57 × 10^−7^, *d* = 0.43; grasp *T*_(586)_ = 7.49, *p* = 2.41 × 10^−13^, *d* = 0.62) (Fig. 2). In contrast, we found no change in reach (*T*_(642)_ = 1.26, *p* = 0.21, *d* = 0.099) or grasp duration (*T*_(642)_ = −1.36, *p* = 0.17, *d* = −0.11) with the left, “non-paretic hand.” For EMG signals, the pattern of activation was generally well-preserved during movements of the non-paretic arm (Fig. 3). However, changes were apparent in some muscles of the paretic arm during reach and grasp after inactivation (Fig. 3*A*), and there was a significant decrease of EMG amplitude in this arm (Fig. 3*B*; paired *t* test: aligned on Grasp Onset, *T*_(12)_ = −4.129, *p* = 0.0014, *g* = −0.44; aligned on Grasp Offset, *T*_(12)_ = −5.985, *p* = 6.36 × 10^−5^, *g* = −0.85). In addition, no activity was observed in the “resting” arm (i.e., opposite to the one performing trials) before or after inactivation. Thus, changes of neuronal activity after inactivation cannot be explained by the appearance or disappearance of covert, “mirror like” EMG activity in the resting arm. After the Post 0.5 h recording session, the monkeys were brought back to the home cage, where they could move freely, with unlimited access to water.

We monitored the progression and extent of deficits caused by inactivation in additional recording sessions, 3 h and 10 h after the injection of muscimol. After 3 h (Post 3 h), impairments with the right hand were so profound that the monkeys could no longer perform the task. However, they still attempted grasping when presented fruits. In some cases, this revealed a limited ability to move digits and an incapacity to produce a precision grip. In other cases, the paretic hand was completely flaccid. Importantly, in all cases, the monkeys attempted to reach to the fruit and eat it with no obvious proximal arm or orofacial movement deficits. This confirmed that the inactivation accurately targeted the M1 hand representation and was seemingly limited to this part of the brain in all inactivation experiments selected in the present study. At 10 h post-inactivation (Post 10 h), the deficits were generally much milder, and they were completely resorbed on the next day.

### The population of neurons recorded in PMv in the control condition

Neuronal data from both monkeys were obtained during 21 recording sessions. For 8 of these sessions, there was no inactivation (Unmanipulated sessions). The 13 others were inactivation experiments. Combining data from the Unmanipulated sessions with data recorded before the injection of muscimol in the inactivation experiments (i.e., session Pre), we identified 520 well-isolated “control” PMv neurons across both hemispheres.

**Figure 4.**
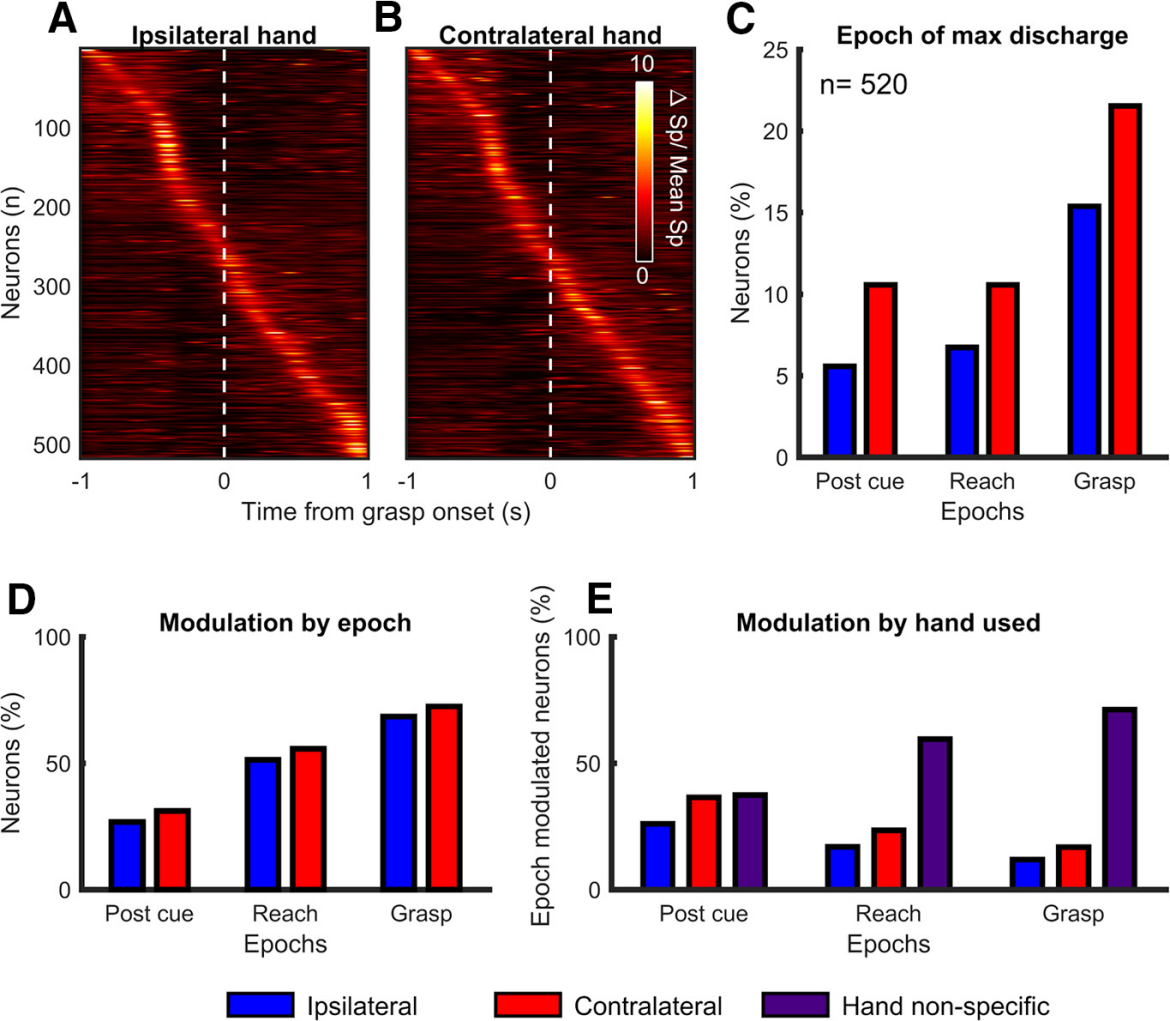
Population of control neurons recorded in PMv. Data from the two hemispheres in both monkeys were pooled for analyses of control neurons. Heat plots of the SDE normalized to the average value in a 2 seconds window around Grasp Onset for all PMv neurons during movements of the ipsilateral (***A***) and contralateral (***B***) hand. Neurons are ordered separately for each hand based on the time of maximal discharge relative to Grasp Onset. Time is in seconds. ***C***, Proportion of neurons with their maximal discharge rate in the various task epochs, during movement of either the ipsilateral or contralateral hand. There is only one count per neuron, across both the task epochs and hand used. There was a greater proportion of neurons with a maximal discharge rate during the grasp epoch and with the contralateral hand. ***D***, Proportion of neurons with significant modulation of discharge rate during the various task epochs (*t* test; *p* < 0.05). Categories are not mutually exclusive. There was a greater proportion of neurons being significantly modulated during the grasp epoch. ***E***, Modulation of neurons during the various task epochs in function of the hand used. For each epoch, only neurons modulated during the use of either hand in that specific epoch are included (sum = 100% per epoch.) Most grasp-related neurons were significantly modulated during movements of both hands.

We characterized the population of neurons recorded by our arrays by determining their preferred epoch and hand (maximal discharge rate), as well as quantifying modulation patterns across epochs (*t* test *p* < 0.05; see Materials and Methods). The population comprised neurons active throughout the various epochs of the task (Fig. 4). However, we found that there was a greater proportion of neurons with a maximal discharge rate (36.9% across both hands; Fig. 4*C*) or being significantly modulated (82.3% across both hands; Fig. 4*D*) during the grasp epoch. This was expected since the arrays were implanted in the region of PMv where the hand representation and neurons contributing to grasp are typically found (Tanji et al., 1988; Schmidlin et al., 2008). Neurons that were not modulated during any of these epochs of interest (9.4% across both hands) often showed modulation after the end of grasp or between trials, several seemingly related to orofacial movements, such as chewing the reward.

Most grasp-related neurons were significantly modulated during movements of both hands (i.e., hand non-specific; 71.3%; Fig. 4*E*). Fewer neurons were modulated during movements of the contralateral hand only (16.8%) and fewer still during movements of the ipsilateral hand only (11.9%). Together, these results demonstrate that a large proportion of PMv neurons recorded from our arrays were involved in grasping, and were active during movements of both hands, albeit with a slight predominance of modulation with movements of the contralateral hand. These findings are very much in line with previous reports of neuronal activity in PMv during hand movements while performing comparable tasks (Rizzolatti et al., 1988; Tanji et al., 1988; Umilta et al., 2007; Michaels and Scherberger, 2018).

### The impact of inactivation on the pattern of modulation of all recorded neurons

**Figure 5.**
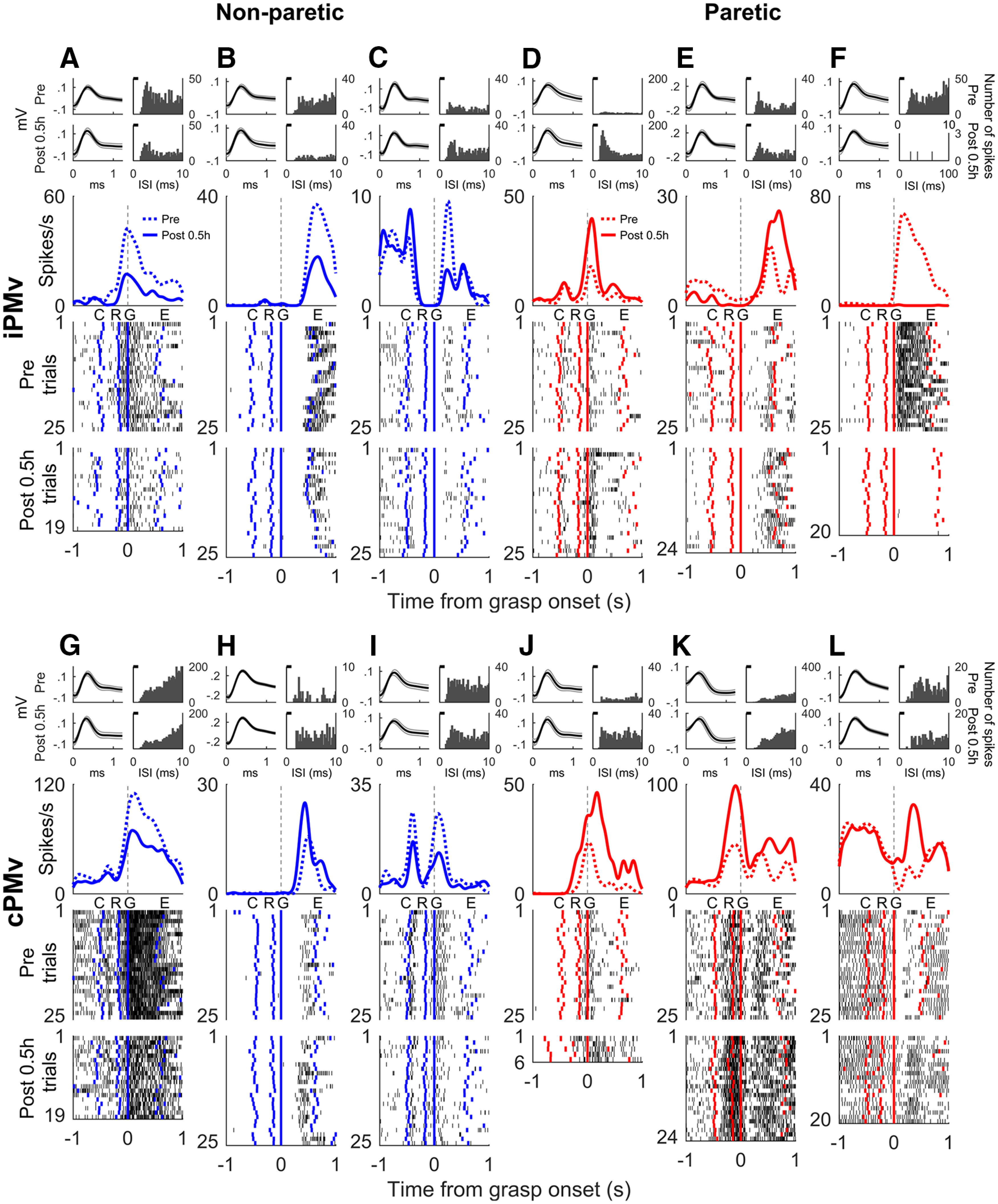
Example neurons with altered pattern of discharge after inactivation. Neurons recorded in iPMv (***A–F***) and cPMv (***G–L***) during movements of the non-paretic (left, blue) and paretic hand (right, red). For each neuron, the top panel shows the average spike shape (±SD with gray shading; left) and the interspike interval count histogram (right), before (top) and after inactivation (bottom). Below are the SDE curves and finally the raster plot of individual trials before (Pre) and 0.5 h after inactivation (Post 0.5 h). Data are aligned on Grasp Onset (time = 0), and time is in seconds. Colored markers in the raster plots represent the timing of other events in each trial (C, GO cue; R, Reach Onset; G, Grasp Onset; E, Grasp Offset). While many neurons were affected in both hemispheres, there was a great variability in the nature of the changes observed in each area.

After muscimol injection, we identified 293 well-isolated post-inactivation neurons, 138 in iPMv and 155 in cPMv. The inactivation of M1 in the left hemisphere induced changes of neural activity in both hemispheres that were readily observed in individual neurons (Fig. 5). After inactivation, both decreases and increases of neural discharge rates were observed. In extreme cases, neurons completely stopped discharging during movements (e.g., Fig. 5*F*), or neurons with little activity started to discharge large bursts (e.g., Fig. 5*L*). The most pronounced changes were often observed in the grasp epoch, suggesting that neuronal activity was particularly affected during movements of the digits.

We compared the entire population of control neurons to the population of post-inactivation neurons (i.e., not limited to within-neuron analyses). To verify if the inactivation had an effect on PMv neurons when the animals were at rest, we looked at the firing rate during the pre-cue epoch, when the hand was at the home plate. We found that the discharge rate during the pre-cue epoch was similar in both hemispheres and regardless of which hand would be used, and that there were no interaction effects between these factors (three-way ANOVA *p* < 0.05; see Materials and Methods). However, the firing rate of PMv neurons was significantly lower after inactivation (*F*_(1,1618)_ = 14.4, *p* = 0.0002, η*_p_*^2^ = 0.0088). These results support that the inactivation resulted in a general decrease of neuronal activity in both hemispheres when the monkeys were not actively involved in the task.

**Figure 6.**
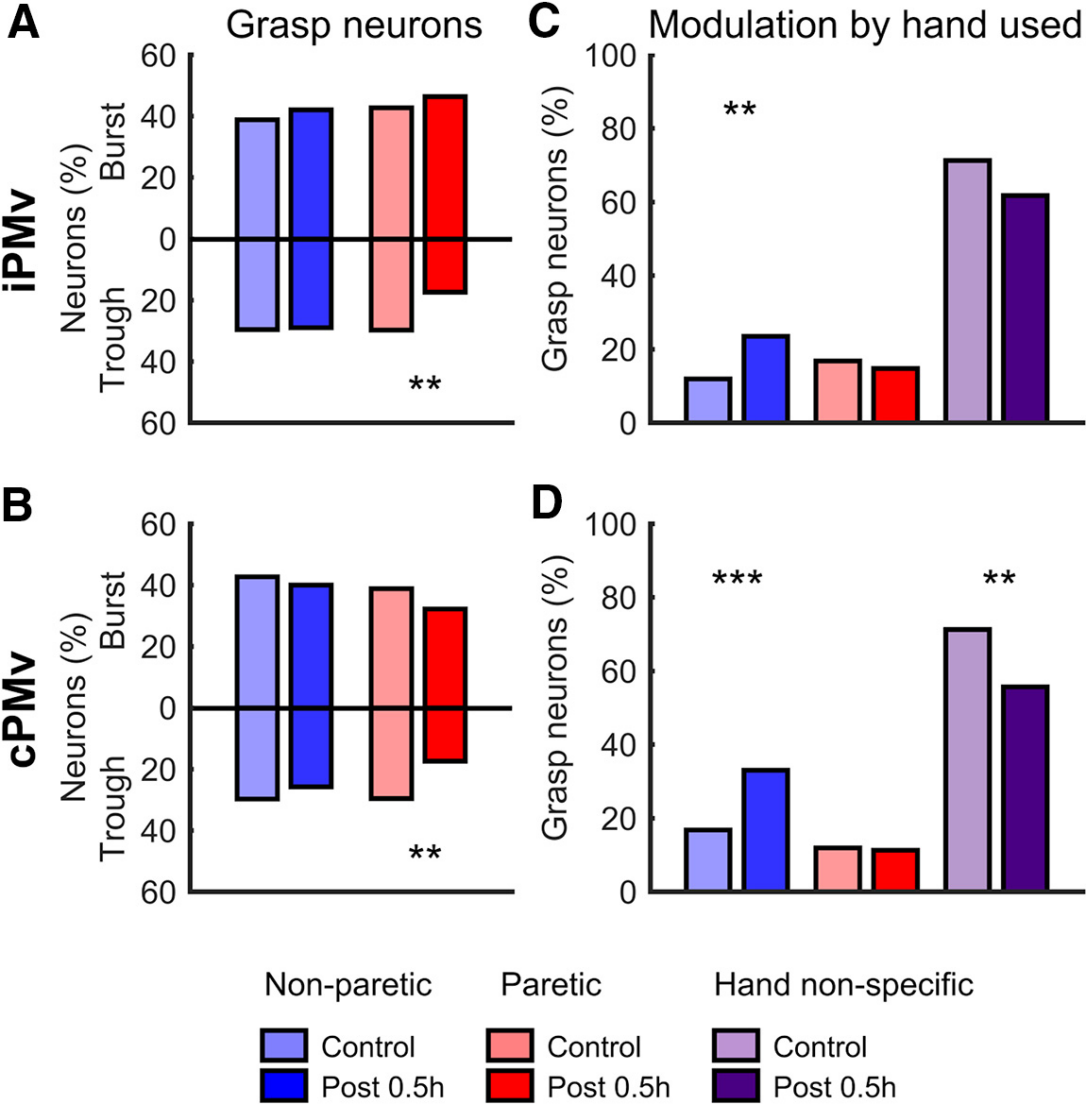
Changes across the PMv neuronal population after inactivation. ***A***, ***B***, Proportion of neurons modulated during grasp (*t* test < 0.05) in iPMv (***A***) and cPMv (***B***) during movement of the non-paretic (blue bars) and paretic hand (red bars). The proportion of neurons with increased discharge rates during grasp, or bursts, is shown above the *x* axis, and the proportion of neurons with decreased discharge rate, or trough, is below. After inactivation, there was a lower proportion of neurons with trough during grasping with the paretic hand in both hemispheres. ***C***, ***D***, Proportion of neurons modulated in function of the hand used in iPMv (***C***) and cPMv (***D***). After inactivation, there was a higher proportion of neurons modulated only during movements of non-paretic hand (blue bar) in both hemispheres and a decrease of neurons modulated during grasp with either hand (i.e., “hand non-specific”; purple bar) in cPMv. **p < 0.01. ***p < 0.001.

The M1 inactivation also affected the proportion of neurons modulated during grasp with the paretic hand in both iPMv (χ^2^_(2,_ *_N_*_= 658)_ = 9.36, *p* = 0.0093, *V* = 0.084) and cPMv (χ^2^_(2,_ *_N_*_= 675)_ = 19.76, *p* = 5.11 × 10^−5^, *V* = 0.12) (Fig. 6*A*,*B*). When looking at the type of neurons affected, we found little difference in the proportion of neurons with a significant burst during grasp. Instead, neurons with a decrease of firing rate, or trough, during grasp were primarily affected (iPMv −12.4%, Bonferroni-corrected *z* test, *z* = −2.91, *p* = 0.0071, *r* = −0.22; cPMv −12.2%, *z* = −3.01, *p* = 0.0053, *r* = −0.22). Finally, the inactivation affected the selectivity of neurons to the hand moved in both the iPMv (χ^2^_(2,_ *_N_*_= 543)_ = 9.85, *p* = 0.0073, *V* = 0.095) and cPMv (χ^2^_(2,_ *_N_*_= 543)_ = 15.05, *p* = 5.39 × 10^−4^, *V* = 0.12) (Fig. 6*C*,*D*). Compared with controls, the post-inactivation population had a greater proportion of neurons only modulated during use of the non-paretic hand in both iPMv (11.6%; *z* = 3.14, *p* = 0.0051, *r* = 0.36) and cPMv (16.2%; *z* = 3.84, *p* = 3.65 × 10^−4^, *r* = 0.37). It also had a lower proportion of hand non-specific neurons that was significant in cPMv (−15.6%; *z* = −3.18, *p* = 0.0043, *r* = −0.17). A similar trend was observed in the iPMv, although it did not reach significance (−9.5%; *z* = −1.96, *p* = 0.15, *r* = −0.1). This suggests that the lower proportion of neurons with trough during grasp with the paretic hand may be largely due a loss of neurons broadly tuned to movements of either hand (i.e., hand non-specific) that became only active during grasp with the non-paretic hand.

### Increases and decreases of firing rate in individual neurons during grasp

The greatest strength of our data are that the continuous recording before and after inactivation allowed us to quantify changes within individual neurons that remained well-isolated throughout any given inactivation experiment (within-neurons analyses; 94 in iPMv and 138 in cPMv). The rest of our analyses were focused on this subpopulation of neurons.

**Figure 7.**
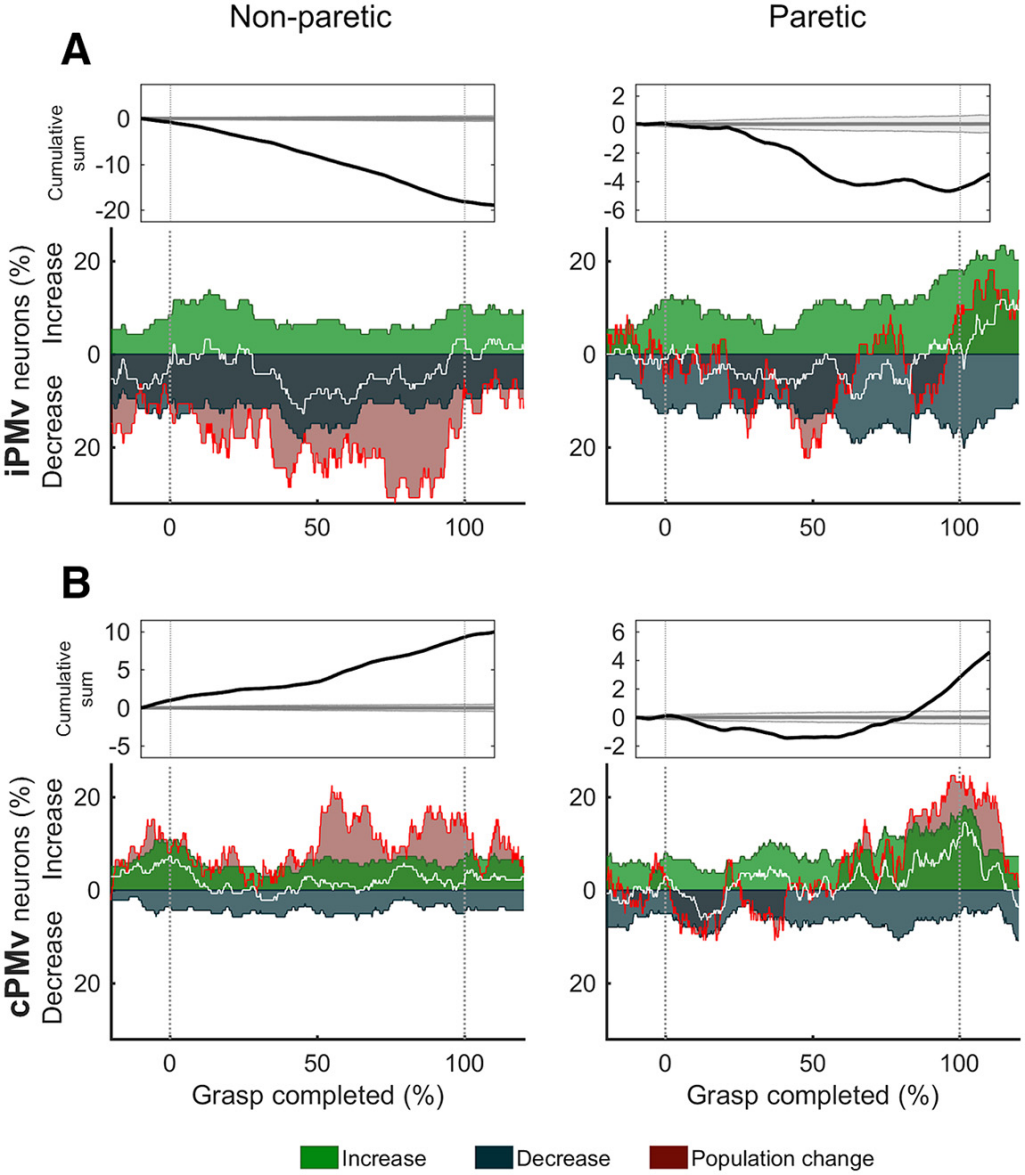
Incidence of neurons with increases and decreases of discharge rate during grasp. Changes of firing rate in iPMv (***A***) and cPMv (***B***) across normalized time during grasp with the non-paretic hand (left column) and the paretic hand (right column). All clearly isolated neurons before and after inactivation are included in this analysis, independently of their tuning to the task epochs (*n* = 94 in iPMv; *n* = 138 in cPMv). In each panel, top plots represent the cumulative sum of the difference between the total population of neurons with increases and decreases of firing rate across time bins (black trace) with the mean and 95% quantile of randomly simulated values (gray line and shadow). Bottom color plots represent the percentage of neurons with “significant” (unpaired *t* tests with *p* < 0.05; see Materials and Methods) increases (green) or decreases (gray-blue) in discharge rate at each moment during grasp. White trace represents the difference in the proportion of all clearly isolated neurons with “significant” increases and decreases of activity. Red shaded area and line represent the same difference but using all neurons, including those with “nonsignificant” changes. In iPMv, more neurons showed decreased discharge rate after inactivation. This population accumulated with time during grasp, in particular with the non-paretic hand. In cPMv, more neurons showed decreased discharge rate after inactivation. This population accumulated with time during grasp, in particular with the paretic hand. 0 = Grasp Onset; 100 = Grasp Offset.

We quantified the incidence of neurons with increases or decreases of neural discharge rate at each moment in time throughout the grasp epoch (Fig. 7). Changes in both iPMv and cPMv, with movements of either hand, were highly heterogeneous. While many neurons had increased firing rates, many others decreased their activity, perhaps to counterbalance each other. Nevertheless, specific and different general trends were present in the two hemispheres. In iPMv, a greater proportion of neurons decreased firing rate after inactivation, in particular when monkeys used the non-paretic hand. In contrast, a greater proportion of neurons increased discharge rate in cPMv, and the largest changes occurred at the end of grasp with the paretic hand. These effects were clearly outside the realm of what could be expected by chance, when compared with Monte Carlo simulated population changes (see Materials and Methods). We confirmed that this increased neuronal activity during movement of the paretic arm was not associated with the appearance of covert EMG activity in the non-paretic arm (see Fig. 3).

### Changes of peak timing in individual neurons following inactivation

**Figure 8.**
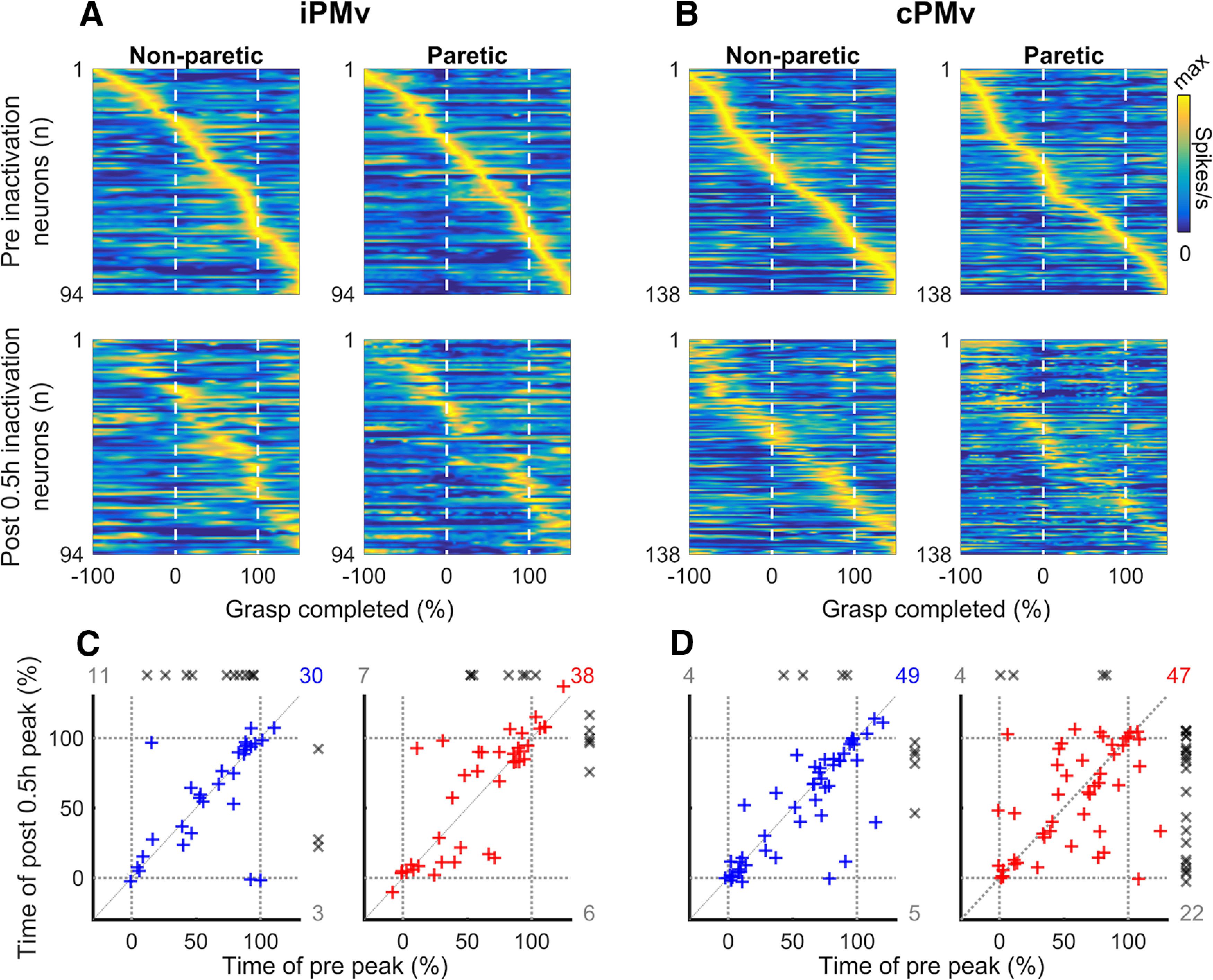
Changes in the timing of peak discharge during grasp after inactivation. ***A***, ***B***, Heat plots of the SDE during grasp for all iPMv (***A***) and cPMv (***B***) neurons well isolated both before and after inactivation (within-neuron analysis) in normalized time. Left and right panels represent activity during movements with the non-paretic and paretic hand, respectively. To emphasize timing shifts over changes in firing rate, the discharge rate of each neuron across time is normalized to its peak value. Neurons are ordered based on the time of their peak discharge relative to Grasp Onset before inactivation (Pre; top row), and the order is kept constant 0.5 h after the inactivation (Post 0.5 h; bottom row). 0 = Grasp Onset; 100 = Grasp Offset. ***C***, ***D***, Peak discharge time of iPMv and cPMv neurons with clear discharge burst during grasp before inactivation (“Pre-Peak,” *x* axis) and/or after inactivation (“Post-Peak,” *y* axis) in normalized time. Colored symbols (+; blue = non-paretic hand; red = paretic hand) and numbers are for neurons with detectable bursts (>1 SD increase from pre-cue epoch) before and after the inactivation. Additional well-isolated neurons that either stopped or started having a detectable burst during grasp after inactivation are plotted along the axes (gray x symbols and numbers). The inactivation affected the temporal pattern of neuronal discharge in both iPMv and cPMv, more so during use of the paretic hand. In iPMv (***C***), many well-isolated neurons with a burst during grasp lost this burst after inactivation. When considering the entire population of neurons that had detectable burst during grasp, either before and/or after inactivation (*n* = 44 for the non-paretic arm and *n* = 51 for the paretic arm), a total of 18 neurons (18.9%) lost this burst after inactivation (*n* = 11 for the non-paretic arm and *n* = 7 for the paretic arm; gray x symbols and count on top of plots). Thus, the loss of modulated neurons was greater during movements of the non-paretic arm (11 of 44; 25.0%). ***D***, In cPMv, many neurons that did not have peaks during grasp before inactivation had one after inactivation. Out of the entire population (*n* = 58 for the non-paretic arm and *n* = 73 for the paretic arm), 27 neurons (20.6%) showed a new burst of activity after inactivation and many more during movements of the paretic arm (22 of 73; 30.1%).

We investigated the effects of M1 inactivation on the timing of peak discharge of iPMv and cPMv neurons during grasp. When looking at all clearly isolated neurons before and after inactivation (Fig. 8*A*,*B*), we found that timing was perturbed for many neurons in both hemispheres, and these changes were greater during paretic hand movements. We quantified the changes of peak discharge timing for neurons with a clear burst during grasp (>1 SD increase from pre-cue epoch) and found a similar pattern of reorganization with this subpopulation (Fig. 8*C*,*D*). In both iPMv and cPMv, several neurons showed altered peak discharge time, and more so during movements of the paretic hand. We interpret this as a desynchronization of PMv neurons' activity in relation to the various components of grasping movements after inactivation, that simultaneously occurs in both hemispheres. Interestingly, many well-isolated neurons in iPMv with a burst during grasp lost this burst after inactivation (*n* = 18, 18.9%; Fig. 8*C*), and more so during movements of the non-paretic hand (*n* = 11, 25.0%; left). In contrast, many neurons in cPMv that did not have peaks during grasp before inactivation had one after inactivation (*n* = 27, 20.6%; Fig. 8*D*), and more so during movements with the paretic hand (*n* = 22, 30.1%; right).

**Figure 9.**
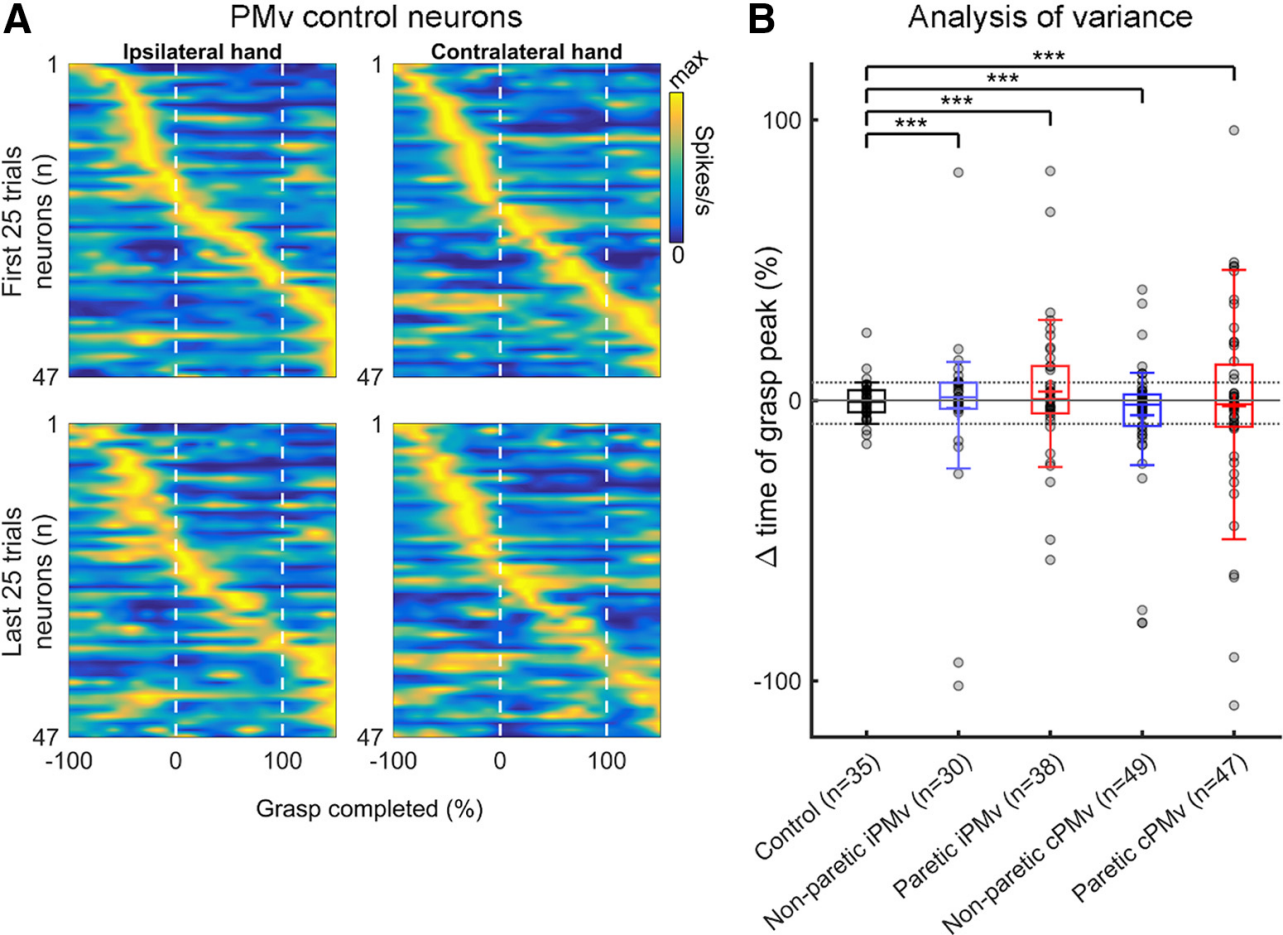
Comparison of variability for the timing of peak discharge in an unmanipulated session. ***A***, We selected the longest unmanipulated session during which we recorded continuously (duration ∼1 h) and identified all well-isolated units in PMv of both hemispheres. As in Figure 8*A*, *B*, the heat plots represent the SDE of these control neurons during the first 25 trials (top) and last 25 trials (bottom). Left and right plots represent data during trials with the ipsilateral and contralateral hand, respectively. Peak discharge timing was largely preserved across control neurons. 0 = Grasp Onset; 100 = Grasp Offset. ***B***, Box-and-whisker plot of the change of peak discharge time of PMv neurons with clear discharge burst during grasp. The plot compares PMv neurons from the same unmanipulated recording session (black) to neurons recorded in iPMv and cPMv in inactivation experiments (i.e., from Fig. 8*C*,*D*). The variance of peak discharge time was significantly greater after inactivation, for both iPMv and cPMv neurons, and this was true during movements of the non-paretic (blue) and paretic arm (red). ***p < 0.001.

To give a better appreciation of the effect of the inactivation on the timing of peak discharge, we compared the effect of the inactivation with the impact of time during a control session that lasted ∼1 h. When comparing the peak discharge time of all the well-isolated neurons at the beginning and the end of that recording session (Fig. 9*A*), the timing of discharges appeared much more stable than what we found after inactivation. Using neurons with a clear burst during grasp, we compared the changes of peak discharge time for these control neurons to the ones caused by inactivation (i.e., neurons from Fig. 8*C*,*D*). The variance of peak discharge time was significantly lower in the control session (Bartlett's statistic χ^2^_(4,_ *_N_*_= 199)_ = 70.53, *p* = 1.76 × 10^−14^). This was true for iPMv neurons during movements of the non-paretic and paretic arm (*post hoc* two-sample *F* tests *F*_(34,29)_ = 0.053, *p* = 4.16 × 10^−13^ and *F*_(34,37)_ = 0.081, *p* = 7.29 × 10^−11^, respectively), and for cPMv also during movements of the non-paretic and paretic arm (*F* tests *F*_(34,48)_ = 0.11, *p* = 1.53 × 10^−9^ and *F*_(34,46)_ = 0.041, *p* = 1.08 × 10^−15^, respectively).

Overall, it seems that M1 inactivation led to a profound alteration of peak discharge timing in PMv that was generalized to both hemispheres, and during grasping with the non-paretic and the paretic arm. These circuit-wide changes also involved the disappearance of discharge bursts for many neurons in iPMv and the emergence of novel discharge bursts for many neurons in cPMv.

### Changes of peak amplitude at the time of peak discharge of individual neurons

**Figure 10.**
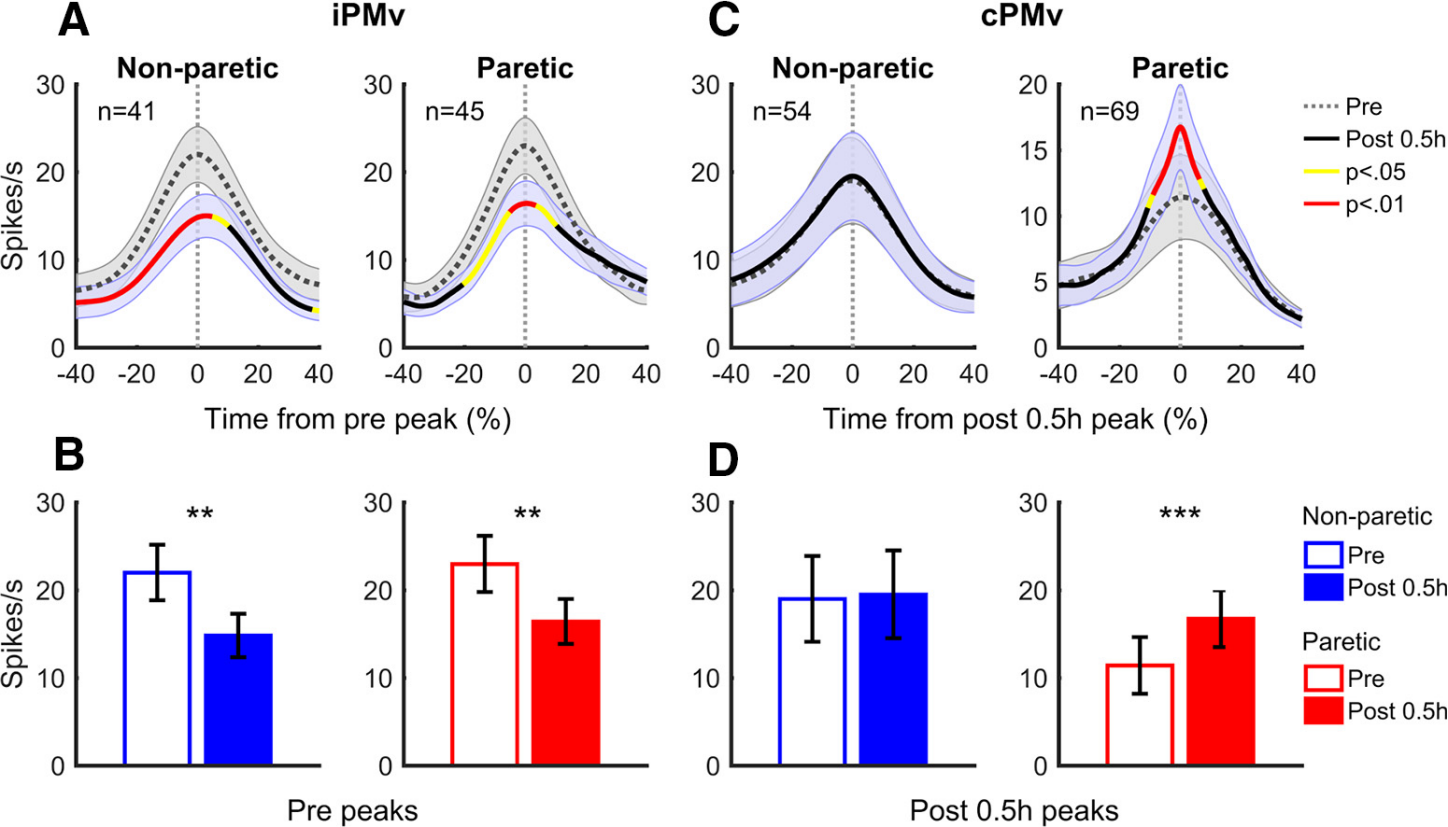
Changes induced by inactivation at the time of peak discharge. ***A***, Average spike density of iPMv neurons during grasp before (dotted line; gray shade represents SEM) and after inactivation (solid line; blue shade represents SEM), when the activity is aligned to the time of peak discharge before inactivation, in normalized time. Colors on the solid line report time bins for which the average firing rate of the population was “significantly” different after inactivation (yellow represents paired *t* test *p* < 0.05; red represents paired *t* test *p* < 0.01). There was a decrease of activity during grasp that was more pronounced during movements of the non-paretic hand (left). ***B***, Comparison of average spike discharge rate of iPMv neurons (+/- SEM) before and after inactivation (paired *t* test at time 0). There was a significant decrease of peak firing rate with movements of both the non-paretic (left; blue) and paretic hand (right; red). ***C***, Average discharge rate of cPMv neurons, when the activity is aligned to the time of peak discharge after inactivation. There was a sharp increase of activity during movements of the paretic hand (right). ***D***, There was a significant increase of peak firing rate with movements of the paretic hand at the time of maximal discharge during grasp (right; red). **p < 0.01. ***p < 0.001.

To consider both firing rate and peak timing, we looked at firing frequency at the time of peak discharge of individual neurons (Fig. 10). For iPMv, given the large count drop of neurons with a detectable peak after inactivation, we identified peak discharge time during grasp before inactivation for each neuron and compared the discharge rate at this time before and after inactivation. This time alignment highlights that iPMv neurons had a large decrease of activity at the time during grasp when they were most active before inactivation. These changes were greater during movements with the non-paretic hand (Fig. 10*A*). When comparing the firing rate at the time of peak discharge (Fig. 10*B*), there was a significant decrease during movements of the non-paretic (*T*_(40)_ = −3.25, *p* = 0.0023, *d* = −0.51) and the paretic hand (*T*_(44)_ = −2.94, *p* = 0.0052, *d* = −0.44). No such decrease was observed in cPMv using this time alignment (*p* > 0.05).

In cPMv, because many neurons started to have significant bursts only after inactivation, we identified the peak discharge time of each neuron after inactivation and compared the discharge rate at this time before and after inactivation. While there was no change when monkeys moved the non-paretic hand, there was a sharp increase of maximal discharge rate during grasp with the paretic hand (Fig. 10*C*). This time alignment highlights that, during movements with the paretic hand, cPMv neurons had increased discharge or new bursts at a time during grasp when they were much less active before inactivation. Peak firing frequency of cPMv neurons was unaffected during movement with the non-paretic hand (Fig. 10*D*; *T*_(53)_ = 0.55, *p* > 0.05, *d* = 0.074) and was significantly increased during grasp with the paretic hand (*T*_(68)_ = 6.37, *p* = 1.90 × 10^−8^, *d* = 0.77). Using this timing alignment, no such increase was observed in iPMv (*p* > 0.05).

Although these population changes were supported by neurons that either lost their discharge peaks (i.e., in iPMv) or had new ones (i.e., in cPMv), the same trends were observed when only looking at neurons that had detectable peaks before and after inactivation. Within this subpopulation of neurons, there was also a decrease of activity in iPMv during grasp with the paretic (−6.15 spikes/s; *T*_(37)_ = −2.37, *p* = 0.023, *d* = −0.32) and the non-paretic hand (−5.69 spikes/s; *T*_(29)_ = −3.1, *p* = 0.0043, *d* = −0.38). In cPMv, there were no changes during movements of the non-paretic hand (*p* > 0.05), but a sharp increase of activity during movements of the paretic hand (4.06 spikes/s; *T*_(46)_ = 3.97, *p* = 2.49 × 10^−4^, *d* = 0.58).

Together, these findings confirm that two very different phenomena occurred simultaneously in iPMv and cPMv after inactivation. Neurons in iPMv decreased firing at the time when they were most active and neurons in cPMv started bursting at a time when they were less active before inactivation.

### Progression of altered neuronal discharge pattern with time after muscimol injection

Finally, we wondered whether the changes in neural activity observed at the onset of impairments would increase along with time and the progression of motor deficits after the muscimol injection. We tracked a subset of electrodes with stable recordings across Post 0.5 h, 3 h, and 10 h sessions (i.e., consistently well-isolated and similar spike shape), and thus that we could reasonably assume to come from the same neurons. In addition, these neurons all had their maximal peak discharge during grasp with the non-paretic hand before inactivation (*n* = 11 iPMv, 34 cPMv neurons). We focused on the non-paretic hand since the monkeys were unable to perform the task with the paretic hand at Post 3 h (see above).

**Figure 11.**
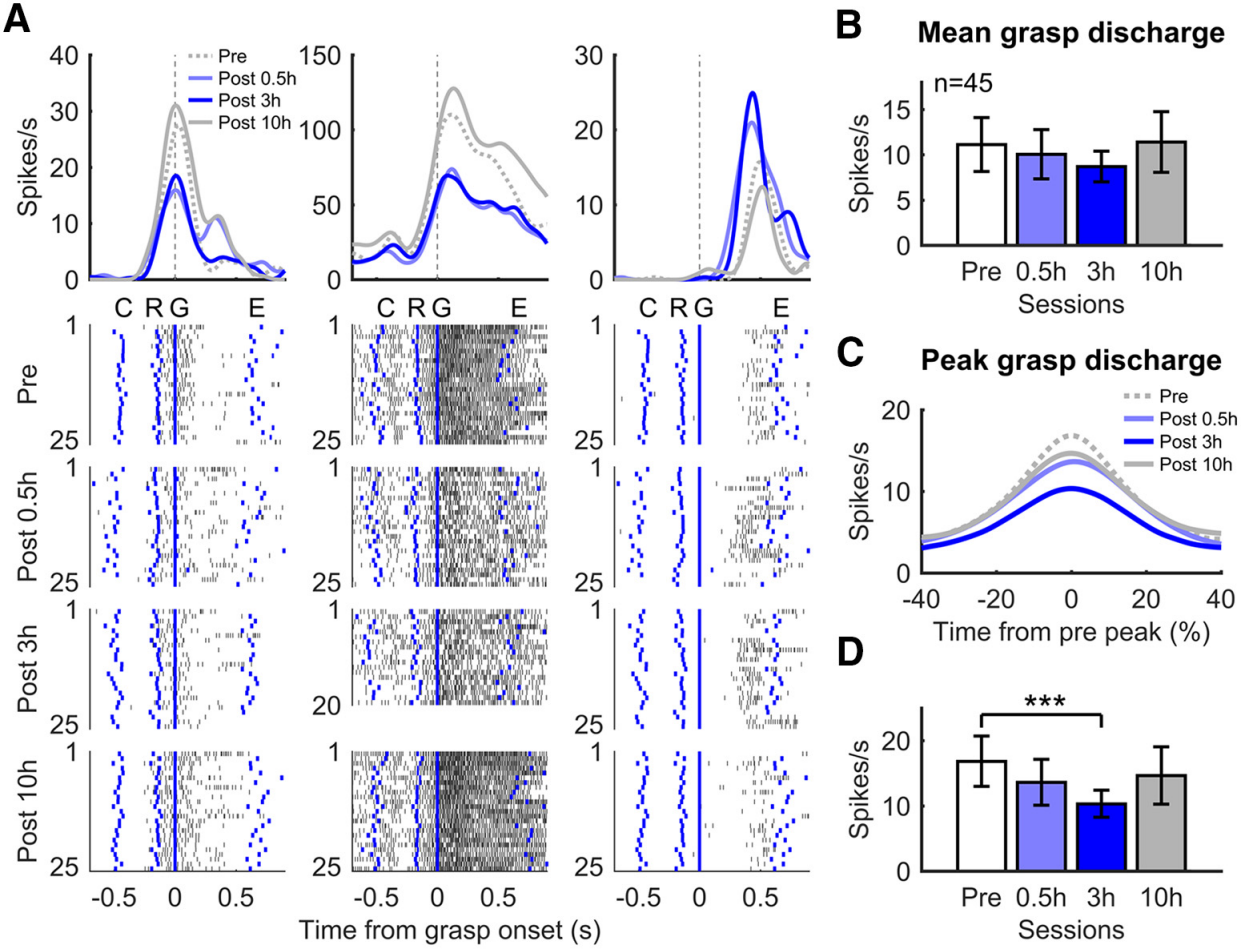
Progression of neuronal changes in PMv with time after muscimol injection. ***A***, Examples of well-isolated neurons, showing decreases (left and middle column) and increases (right column) of activity during use of the non-paretic hand in real time (seconds). There is a clear impact of the inactivation on neural activity at time Post 0.5 h and Post 3 h. At time Post 10 h, the firing rate tended to return toward pre-inactivation values. ***B***, Average firing rate (+/- SEM) during grasp. The neuronal population showed a similar trend across different data collection time points. The largest change from baseline was observed at Post 3 h, with a return toward baseline at Post 10 h. ***C***, Profile of peak discharge of PMv neurons at Post 0.5 h, Post 3 h, and Post 10 h, when aligning data using the time of peak maximal discharge of each neuron before inactivation in normalized time (Pre-Peak). The average firing rate progressively decreased at Post 0.5 h and Post 3 h and then came back toward pre-inactivation values at Post 10 h. ***D***, Maximal peak discharge rate (±SEM) using Pre-Peak time alignment. There was a significant decrease at Post 3 h. At Post 10 h, neural activity seemed to have recovered, returning to Pre-inactivation levels. ***p < 0.001.

Looking at individual neurons, we found examples with increases and with decreases of activity following inactivation (Fig. 11*A*). We quantified the mean spike firing rate during grasp of the neuronal population and compared the different data collection sessions. Since we did not observe a main effect of area (iPMv, cPMv) nor interaction between area and session (Pre, Post 0.5 h, 3 h, 10 h) (repeated-measures two-way ANOVA; *F*_(3,129)_ = 0.67, *p* = 0.57, η*_p_*^2^ = 0.013), we merged the neuronal populations from the two hemispheres. With this analysis, there was a trend of a progressive decrease of firing rate for Post 0.5 h and Post 3 h sessions and a return toward baseline for Post 10 h (Fig. 11*B*). However, the main effect of session was not significant (*F*_(3,132)_ = 1.84, *p* = 0.14, η*_p_*^2^ = 0.04). We then compared the discharge amplitude of the population at the time of their maximal discharge before inactivation (Fig. 11*C*,*D*). Again, since we did not observe any significant interaction between area and session (*F*_(3,129)_ = 0.52, *p* = 0.66, η*_p_*^2^ = 0.012), we merged the two populations. With this analysis, there was a significant effect of session (*F*_(3,132)_ = 4.21, *p* = 0.007, η*_p_*^2^ = 0.087). While there was no difference between Pre and Post 0.5 h (Bonferroni-corrected paired *t* test; *T*_(44)_ = −1.72, *p* = 0.08, *d* = −0.26), there was a significant decrease in peak neural activity at Post 3 h (*T*_(44)_ = −3.49, *p* = 6.67 × 10^−4^, *d* = −0.52), when behavioral deficits were most pronounced. At Post 10 h, neural activity seemed to have recovered, returning to Pre-inactivation levels (*T*_(44)_ = −1.17, *p* = 0.24, *d* = −0.17). These analyses support that the changes of neuronal activity at Post 0.5 h reflect the level impairments and that they likely progressed in a similar manner, becoming more pronounced as behavioral deficits worsened.

## Discussion

We investigated the impact of a cortical lesion in M1 on neuronal activity of PMv in the ipsilesional and contralesional hemispheres while monkeys produced grasping movements. To do so, we used reversible inactivation of M1 with an agonist of the neurotransmitter GABA, muscimol. GABA is ubiquitously present in the neocortex, and cortical injections of muscimol lead to hyperpolarization of all neuron types (Matsumoto, 1989; Martin and Ghez, 1999). The discharge rate of these neurons is profoundly reduced or completely abolished (Hess and Murata, 1974), causing hypometabolism in the affected region (Martin, 1991). Several experiments support that the behavioral effects of muscimol inactivation are comparable to the ones observed acutely after permanent brain lesions. For example, muscimol inactivation or permanent lesion in the motor cortex can both cause movement trajectory errors, impaired dexterous hand control, and loss of independent finger movements (Martin and Ghez, 1993; Hoffman and Strick, 1995; Schieber and Poliakov, 1998; Brochier et al., 1999; Nudo et al., 2003; Hoogewoud et al., 2013). Muscimol is thus considered an acceptable tool to study the acute effects of injuries in the brain.

In the present study, we favored reversible inactivation techniques over more clinically relevant brain injury models to continuously record isolated neurons before and after the onset of motor deficits. This powerful approach helped us reveal that extensive and complex neuronal reorganization takes place in both hemispheres, at the very onset of behavioral impairments. We view this rapid reorganization as a consequence of the disruption of the motor network's homeostasis (von Monakow, 1914; Carrera and Tononi, 2014). We thus expect that changes of comparable magnitude simultaneously take place in other brain regions interconnected with the site of injury. Because of the very short delay after injury, molecular and cellular processes specific to the type of injury (e.g., traumatic or ischemic, etc.) probably have little impact on neuronal reorganization at this point but instead become involved later during recovery. Accordingly, the changes we describe should have many common features with the ones evoked acutely after any type of lesion in the brain.

### Bihemispheric detuning of neuronal activity in PMv during grasp with the paretic hand

It is well known that some PMv neurons pause during movements of the hand (Tanji et al., 1988). After inactivation of M1, we found that trough neurons in both hemispheres were more likely to lose their tuning to grasp with the paretic hand. In the oculomotor system, inhibitory interneurons are tonically active to prevent unwanted movements. When they pause, the release of inhibition favors the firing of excitatory neurons driving motor outputs (Evinger et al., 1982; Paré and Guitton, 1998). One possibility is that trough neurons in PMv serve a comparable purpose. If so, their loss in iPMv after brain injury could favor the generation of undesirable co-contractions and impair individuated finger movements (Lang and Schieber, 2004). The presence of such a gating mechanism in the premotor cortex is, however, uncertain (Kaufman et al., 2010). In cPMv, the decrease of hand nonspecific neurons and the increase of neurons modulated with movement of only the non-paretic hand could support compensatory behavior with the non-paretic hand (Jones, 2017).

A conspicuous impact of inactivation was the alteration of discharge burst timing during grasp. These changes were also observed in iPMv and cPMv, and more pronounced with movements of the paretic hand. Similarly, stroke in the somatosensory cortex induces chronic impairments in the temporal fidelity of responses to peripheral stimulation (Sweetnam and Brown, 2013), which could be because of a dysfunction of the corticothalamic feedback circuit involved in the inhibition of thalamocortical neurons (Paz et al., 2010). In the visual system, corticothalamic feedback from the visual cortex to the lateral geniculate nucleus is involved in spike-timing precision of thalamic neurons' responses to incoming visual signals (Hasse and Briggs, 2017). It is thus possible that changes in the timing of neuronal activity in PMv during grasp are because of the loss of corticothalamic signals from M1 that indirectly affect inputs to PMv. Alternatively, timing abnormality could be caused by the loss of feedback of cortical projections from M1 to iPMv and cPMv (Dancause et al., 2007; Hamadjida et al., 2016). In any case, our results suggest that brain injuries immediately have a major impact on the timing of neuronal activity in spared cortical areas, across the bihemispheric network. The restoration of proper timing or retuning of the neuronal discharges in these areas may thus be an important contributor to recovery. Along these lines, activity-dependent stimulation was used to resynchronize the neuronal activity of spared motor and somatosensory cortex after brain injury in rats (Guggenmos et al., 2013). Remarkably, this precisely timed, closed-loop approach contributed more effectively to recovery than stimulation delivered using arbitrary timing, in an open-loop design.

### Hemisphere-specific alteration of neuronal activity in PMv

The reorganization of iPMv and/or cPMv activity during movement of the paretic hand is one of the most consistent findings across human imaging studies after stroke (Rehme et al., 2012). The neuronal correlate to this metabolic reorganization is, however, largely unknown. Previous invasive studies have shown that cortical injury can induce bihemispheric neuronal reorganization, with a decrease of responses in the ipsilesional and an increase in the contralesional hemisphere (Sigler et al., 2009; Mohajerani et al., 2011). However, these studies have provided little insight into changes that occur in the motor network during active generation of movements and have been essentially limited to rodents. This is further complicated by the equivocal homology between the rostral motor area in rodents and premotor cortex of primates (Rouiller et al., 1993; Touvykine et al., 2020). Our findings clarify these issues by showing rapid and heterogeneous effects of brain injury on neuronal activity in the premotor cortex of nonhuman primates during the generation of hand movements.

Several of our analyses indicate a decrease of activity in iPMv. This decrease might in part explain the reduction of EMG activity observed during the movement of the paretic hand. However, it is worth nothing that iPMv activity was more affected during movements with the non-paretic arm, for which we found no changes of EMG. This suggests that at least some reorganization in iPMv was because of other factors, such as, for example, a disruption of interactions between neurons within iPMv or with cPMv across the hemispheres. In addition, the decreased firing rate of some iPMv neurons could be caused by changes of interactions with other neurons in distant cortical areas of the grasping network (Davare et al., 2011), also likely affected by the injury.

Among ipsilateral premotor areas, PMv has the most numerous projections to M1 (Dum and Strick, 2005) and bears powerful facilitatory effects on M1 outputs (Cerri et al., 2003; Quessy et al., 2016). Interestingly, several studies in monkeys looking at iPMv in the chronic phase of recovery after brain injury have reported profound physiological and anatomic reorganization (Dancause et al., 2005, 2006; Yamamoto et al., 2019). The contribution of iPMv to recovery has also been supported in pharmacological inactivation studies (Murata et al., 2015). If the decrease of neuronal activity in iPMv persists during recovery, it could thus contribute to impairments of movements with the paretic hand. To counter the acute decrease of activity in iPMv, it may be interesting to deliver excitatory neuromodulatory protocols to this area early after stroke, for example using high-frequency repetitive TMS (Pascual-Leone et al., 1994).

Our data also highlight that, in awake monkeys, the increased neuronal activity in the contralesional hemisphere is movement- and effector-specific. After injury, the firing rate in PMv of both hemispheres was actually lower at rest. The neuronal activity in cPMv was only increased with movements of the paretic hand, at the end of grasp. This timing suggests that it occurred while the monkeys attempted to hold the reward and initiate the movement back to the mouth. The novel activity may thus be caused by a mismatch between the predicted and effective movement (Wolpert and Miall, 1996) or the visual detection of end-point errors (Inoue et al., 2016). If this is the case, it is intriguing that such an error signal would be present in cPMv, but not detectable in iPMv. Regardless of its cause, increased neuronal discharges in cPMv could potentially have negative effects on the generation of paretic hand movements. Inversely to iPMv, cPMv exerts strong inhibition on the production of M1 outputs (Quessy et al., 2016; Cote et al., 2020). The application of inhibitory stimulation protocols over cPMv early after brain injury, for example using low-frequency repetitive TMS (Chen et al., 1997), could thus be a valid strategy to help recovery of hand movements. However, given the specificity of neuronal changes in cPMv, perhaps activity-dependent, closed-loop modulation would be more effective. This could be achieved, for example, using disruptive single TMS pulse to this area (Day et al., 1989) at the end of grasp with the paretic hand.

### The impact of rapid neuronal reorganization after brain injury on recovery

Longitudinal imaging studies in both animals and humans tend to support that the initial impact of brain injury is profound, inducing changes across multiple areas in both hemispheres (Dijkhuizen et al., 2003; Rehme et al., 2012). Behavioral recovery is accompanied by a return toward normal functional connectivity in the network and refocusing of activity in the ipsilesional hemisphere, in particular in M1 (Grefkes and Fink, 2014). It should be kept in mind that these correlational studies provide limited information about the functional role of the changes that take place in the brain. Early changes could reflect negative processes that contributed to impairments. Alternatively, they may reflect rapid adjustments in the circuit to compensate for the neuronal loss caused by the lesion and preserve some residual function. Either way and importantly, the magnitude of early hemodynamic changes correlates with impairments (Weber et al., 2008; Rehme et al., 2011; van Meer et al., 2012) and has a predictive value for motor recovery (Marshall et al., 2009; Rehme et al., 2015; Hannanu et al., 2017). Early shifts of neuronal activity can thus affect subacute reorganization and, consequently, recovery.

Despite the general trends in the two hemispheres, one striking feature in our data was the heterogeneity of effects across neurons. This diversity likely reflects the complexity of neuronal processing that takes place in PMv (Rizzolatti and Luppino, 2001). The initial impact of the lesion on a PMv neuron may vary with its function before the lesion and the specific interactions it entertained with the site of injury. This heterogeneity may create an unstable state that is particularly malleable, and that offers a window of opportunity for external manipulation. Supporting this view, inactivation of the contralesional hemisphere in rats increased recovery when initiated rapidly after stroke, but failed to do so when longer delays were used (Mansoori et al., 2014; Dancause et al., 2015). Similarly, in humans after stroke, repetitive TMS seems to be more beneficial when started early after the lesion (van Lieshout et al., 2019). With time after injury, shifts in the neuronal population may become more uniform across the area and stabilize, decreasing the potential impact of treatments. Obviously, the progression of neuronal reorganization after brain injury will have to be investigated to verify these hypotheses. However, our data clearly highlight the complexity of the reorganization triggered by acute brain injuries, at the very onset of motor impairments. A better knowledge of these changes will lead to the elaboration of new hypotheses for the design of neuromodulatory strategies that target specific neuronal mechanisms, in different components of the network, to favor recovery.
